# Learnings about Aβ from human brain recommend the use of a live-neuron bioassay for the discovery of next generation Alzheimer’s disease immunotherapeutics

**DOI:** 10.1186/s40478-023-01511-2

**Published:** 2023-03-10

**Authors:** Zemin Wang, Ming Jin, Wei Hong, Wen Liu, David Reczek, Valentina N. Lagomarsino, Yuan Hu, Tim Weeden, Matthew P. Frosch, Tracy L. Young-Pearse, Laurent Pradier, Dennis Selkoe, Dominic M. Walsh

**Affiliations:** 1 Laboratory for Neurodegenerative Research, Ann Romney Center for Neurologic Diseases, Brigham and Women’s Hospital, Hale Building for Transformative Medicine, 60 Fenwood Road, Boston, MA 02115 USA; 2 Sanofi-Genzyme Corporation, Framingham, MA 01701 USA; 3 Ann Romney Center for Neurologic Diseases, Brigham and Women’s Hospital, Harvard Medical School, Boston, MA 02115 USA; 4 The Brain Cognition and Brain Disease Institute, Shenzhen Institute of Advanced Technology, Chinese Academy of Sciences; Shenzhen-Hong Kong Institute of Brain Science-Shenzhen Fundamental Research Institutions, Shenzhen, Guangdong China; 5 Massachusetts General Institute for Neurodegenerative Disease, Massachusetts General Hospital and Harvard Medical School, Charlestown, MA 02129 USA; 6 Sanofi R&D, 91380 Chilly-Mazarin, France

**Keywords:** Amyloid β-protein, Automated live-cell imaging, Immunotherapy, Long-term potentiation, Monoclonal antibodies, Neuritic dystrophy, Soluble aggregates

## Abstract

Despite ongoing debate, the amyloid β-protein (Aβ) remains the prime therapeutic target for the treatment of Alzheimer’s disease (AD). However, rational drug design has been hampered by a lack of knowledge about neuroactive Aβ. To help address this deficit, we developed live-cell imaging of iPSC-derived human neurons (iNs) to study the effects of the most disease relevant form of Aβ-oligomeric assemblies (oAβ) extracted from AD brain. Of ten brains studied, extracts from nine caused neuritotoxicity, and in eight cases this was abrogated by Aβ immunodepletion. Here we show that activity in this bioassay agrees relatively well with disruption of hippocampal long-term potentiation, a correlate of learning and memory, and that measurement of neurotoxic oAβ can be obscured by more abundant non-toxic forms of Aβ. These findings indicate that the development of novel Aβ targeting therapeutics may benefit from unbiased activity-based discovery. To test this principle, we directly compared 5 clinical antibodies (aducanumab, bapineuzumab,  BAN2401, gantenerumab, and SAR228810) together with an in-house aggregate-preferring antibody (1C22) and established relative EC_50_s in protecting human neurons from human Aβ. The results yielded objective numerical data on the potency of each antibody in neutralizing human oAβ neuritotoxicity. Their relative efficacies in this morphological assay were paralleled by their functional ability to rescue oAβ-induced inhibition of hippocampal synaptic plasticity. This novel paradigm provides an unbiased, all-human system for selecting candidate antibodies for advancement to human immunotherapy.

**Supplementary Information:**

The online version contains supplementary material available at 10.1186/s40478-023-01511-2.

## Introduction

Four decades of research in laboratories and clinics worldwide has yielded evidence that the extracellular accumulation of the amyloid β-protein (Aβ) can trigger neuronal deposition of altered tau proteins and their gradual spread across brain regions serving memory and cognition [49]. Yet there is still only a rudimentary understanding about the forms of Aβ which mediate disease. Studies using synthetic Aβ have defined basic parameters governing toxicity, namely, that Aβ monomers are not toxic, and that toxicity requires active aggregation [57]. The identification of intermediates formed during in vitro aggregation experiments [13, 24, 48] and the demonstration that Aβ secreted from cultured cells was synaptotoxic [46] gave rise to the so-called Aβ oligomer (oAβ) hypothesis [21, 47]. Nonetheless, there remains great confusion about what constitutes an oligomer, and which oligomers are toxic [2]. In the human brain, Aβ is known to exist in many forms. Extensive prior work documented myriad primary structures, including a diversity of N- and C-termini [4, 27, 34] and post-translational modifications [17, 22, 38]. A variety of different types of insoluble deposits have been described [9, 54] and a broad range of soluble aggregates, dimers, and monomers have been detected in aqueous extracts of human brain [4, 11, 37, 40, 42].

Given the widespread interest in Aβ oligomers, it is surprising that more efforts have not been made to characterize and study soluble forms of Aβ isolated from human brain [5, 52]. Previously we showed that the majority of Aβ species extractable from AD cortex are large and inactive [55] and that the most bioactive species are those that can readily diffuse from brain parenchyma [14]. Aging, cellular senescence, the inflammatory milieu, and the presence of agents (e.g., tau) that modulate or synergize Aβ activity complicate the attribution of activity to a single species [31, 36].

We recently developed live-cell imaging of iPSC-derived human neurons (iNs) to study the dynamic effects of human brain-derived Aβ [14, 19], and here we show that activity in this assay parallels disruption of hippocampal long-term potentiation (LTP). Then we used 10 AD brain aqueous extracts to compare the extent of bioactivity with the amounts and forms of Aβ measured using five distinct assays. Consistent with the notion that only a fraction of extractable Aβ is overtly toxic and its measurement is obscured by more abundant less toxic Aβ species and/or agents which modulate or synergize Aβ activity, we observed no relationship between the amount of any measured form of Aβ and presence or extent of neuroactivity. Our results indicate that the development of Aβ-targeting therapeutics would be best guided by activity rather than structural based discovery. Moreover, the natural cocktail of Aβ sequences and aggregation states present in AD brain extracts provides a stringent selection for active agents that discriminate between disease-relevant and irrelevant forms of Aβ.

Armed with this information, we compared some leading anti-Aβ antibodies currently or recently in human trials. All six antibodies protected against oAβ-induced neuritotoxicity (i.e., reduction in the number and/or complexity of neurites) by AD brain extracts, but there were readily quantifiable differences between them. When a subset of antibodies was tested in the more labor-intensive LTP paradigm, they dose-dependently protected against Aβ-mediated disruption of hippocampal synaptic plasticity. This rigorous quantitative analysis recommends our all human live-neuron imaging paradigm to screen for beneficial properties of candidate antibodies. The method is technically facile and scalable for medium-throughput screening of many monoclonal antibodies and other reagents that might protect against AD-type neuritic/synaptic impairment.

The most effective treatment for overt symptomatic AD will likely require more than one therapeutic [49], with tau targeting agents being an attractive means to augment anti-Aβ immunotherapy. Nonetheless, recent clinical trials demonstrate that treatment with certain anti-Aβ antibodies is disease modifying [6, 45] raising the prospect that further optimizing this approach may yield even more benefit.

## Materials and methods

### Reagents and chemicals

Aβ1–42 was prepared and purified by Dr. James I. Elliott at the ERI Amyloid laboratory, Oxford, CT, USA. Peptide mass and purity (> 99%) was confirmed by electrospray/ion trap mass spectrometry and reverse-phase HPLC. Peptide standards were prepared, aliquoted and frozen at 10 ng/μL in 50 mM ammonium bicarbonate, pH 8.5 [14]. Gel filtration standards were purchased from Bio-Rad (Hercules, CA). All other chemicals and reagents were of the highest purity available and unless indicated otherwise were obtained from Sigma-Aldrich (St. Louis, MO).

### Antibodies

The antibodies used and their sources are described in Table 1. S97 is a novel pan anti-Aβ rabbit antiserum whose characterization is shown in Additional file 1: Fig. S1. When used for immunodepletion, protein A-purified S97 was conjugated to protein A Sepharose (PAS) beads. Protein A purified pre-immune rabbit serum was conjugated to PAS beads and used as a control, and brain extracts treated with this material are referred to as Mock immunodepletions.

**Table 1:** Primary and secondary antibodies [20, 26, 29, 41]

Antibody	Type	Antigen/epitope	Dilution for IP	Conc. for WB	Conc. For ELISA	Dilution for AT	Conc. for incucyte	Conc. for LTP	Source/reference
266	Monoclonal	Aβ16–23	–	–	3 µg/ml	–	–	–	Elan/Seubert et al. [41]
2G3	Monoclonal	Aβ terminating at Val40	–	1 µg/mL	–	–	–	–	Elan/Johnson-Wood et al. [20]
21F12	Monoclonal	Aβ terminating at Ile42	–	1 µg/mL	1 µg/mL	–	–	–	Elan/Johnson-Wood et al. [20]
1C22	Monoclonal	Aβ aggregates	–	–	3 µg/mL	1: 50	0.5, 1, 1.5, 2 and 3 µg/mL	3 and 5 µg/mL	Walsh lab/Mably et al. [26]
3D6	Monoclonal	Aβ1–5	–	–	1 µg/mL	–	–	–	Elan/Johnson-Wood et al. [20]
AW7	Polyclonal	Pan anti–Aβ	1: 80	–	–	–	–	–	Walsh lab/McDonald et al. [29]
S97	Polyclonal	Pan anti–Aβ	1: 80	–	–	–	–	–	Walsh lab
Aducanumab	Monoclonal	Aβ3–6/Aβ fibril	–	–	–	–	0.5, 1, 1.5, 2 and 3 µg/mL	3 and 5 µg/mL	Sanofi
BAN2401	Monoclonal	Aβ protofibril	–	–	–	–	1.5 µg/mL	–	Sanofi
Bapinezumab	Monoclonal	Aβ1–7/Aβ monomer and fibril	–	–	–	–	0.5, 1, 1.5, 2 and 3 µg/mL	3 µg/mL	Sanofi
Gantenerumab	Monoclonal	Aβ1–11/Aβ oligomer and fibril	–	–	–	–	1.5 µg/mL	–	Sanofi
SAR228810	Monoclonal	Aβ protofibril and fibril	–	–	–	–	0.5, 1, 1.5, 2 and 3 µg/mL	3 and 5 µg/mL	Sanofi
Avastin	Monoclonal	–	–	–	–	–	1.5 µg/mL	3 and 5 µg/mL	Myoderm

### Preparation of aqueous extracts from human brain

Frozen brain tissue was provided by the Massachusetts ADRC Neuropathology Core, Massachusetts General Hospital (Boston, MA), University of Miami Miller School of Medicine (Miami, FL) and Manchester Brain Bank of the Medical Research Council at University of Manchester (Manchester, UK). Brain tissue was obtained from 11 patients who died with mild to moderate AD (Table 2), and was used in accordance with the Partners Institutional Review Board (Protocol: Walsh BWH 2011). Aqueous extracts were prepared as described previously [50]. In brief, 20 g of cortical gray matter was Dounce-homogenized in 5 volumes of ice-cold artificial cerebrospinal fluid base buffer (aCSF-B) (124 mM NaCl, 2.8 mM KCl, 1.25 mM NaH_2_PO_4_, 26 mM NaHCO_3_, pH 7.4) supplemented with protease inhibitors (5 mM ethylenediaminetetraacetic acid (EDTA), 1 mM ethyleneglycoltetraacetic acid (EGTA), 5 μg/mL leupeptin, 5 μg/mL aprotinin, 2 μg/mL pepstatin, 120 μg/mL pefabloc and 5 mM NaF). The resulting homogenates were centrifuged at 200,000 g for 110 min and 4 °C in a SW41 Ti rotor (Beckman Coulter, Fullerton, CA), and the upper 80% of the supernatant was removed and dialyzed against fresh aCSF-B, with 3 buffer changes (once every 24 h over 72 h). Brain extracts were then divided into 2 parts: 1 portion was immunodepleted (ID) of Aβ by 3 rounds of 12 h incubations at 4 °C with the anti-Aβ polyclonal antibody S97 conjugated to PAS beads; the second portion was treated in an identical manner with pre-immune serum conjugated to PAS beads. Extracts depleted of Aβ are referred to as ID-AD, and extracts treated with pre-immune serum are referred to as mock-AD. Samples were cleared of beads, and 0.5 mL aliquots removed to low protein binding Eppendorf tubes (Eppendorf, Hamburg, Germany) and stored at − 80 °C until used. Samples were thawed once and used.

**Table 2:** Demographic details of cases used in this study

ID#	Age	Gender	PMI (h)	Clinical diagnosis	Neuropathology diagnosis	B&B	Thal	CERAD	NIA-AA	Vonsattel
Br. 1	86	F	35	AD	Mild AD & CAA	III	N.A	Moderate	N.A	N.A
Br. 2	90	F	4.5	AD	AD	IV	N.A	N.A	N.A	N.A
Br. 3	93	F	59	Dementia	Moderate AD & Severe CAA	III-IV	N.A	Moderate	N.A	N.A
Br. 4	68	F	36	AD	AD	VI	4	Moderate	N.A	Absent
Br. 5	87	M	18	AD	Mild AD	IV	1	Sparse	A1B2C1	Absent
Br. 6	65	F	18	AD	Mild AD	II	2	Moderate	A1B1C2	3
Br. 7	77	M	26	CAA	Mild AD	III	3	Moderate	A2B2C2	Absent
Br. 8	92	F	5	AD	Mild AD	IV	5	Sparse	A3B2C1	1
Br. 9	89	F	72	AD	Mild AD & CAA	II-III	N.A	Moderate	N.A	N.A
Br. 10	84	F	48	Dementia	AD	III	N.A	Moderate	N.A	N.A
Br. 11	94	F	5	AD	AD	IV	N.A	N.A	N.A	N.A

A summary of all available data is present. Thal and Vonsattel scoring is shown for the 5 cases for which they were available. CERAD scores were available for all but two cases

*F* female, *M* male, *PMI* post-mortem interval, *AD* Alzheimer's disease, *CAA* cerebral amyloid angiopathy, *B&B* Braak and Braak stages, *Thal* Thali amyloid stages, *CERAD* Constortium to Establish a Registry for Alzheimer's Disease scores, *NIA-AA* The National Institute on Aging—Alzheimer's Association Alzheimer's diagnose criteria; Vonsattel grades for CAA

### MSD Aβ immunoassays

Samples were analyzed with and without pre-incubation in 5 M GuHCl, and Aβ was detected by assays preferential for Aβ ending at Val40 or Ala 42, respectively. GuHCl dissociates soluble Aβ aggregates allowing for their detection with these monomer-preferring assays [28]. The x-40 assay uses monoclonal antibodies (mAb) m266 (3 μg/mL) for capture and biotinylated 2G3 (0.2 μg/mL) for detection; the x-42 assay uses m266 (3 μg/mL) for capture and biotinylated 21F12 (0.4 μg/mL) for detection. Briefly, 20 μL of extract was incubated with 50 μL of 7 M GuHCl at 4 °C overnight. Thereafter samples were diluted 1:10 with assay diluent so that the final GuHCl concentration was 0.5 M. To match the buffer composition of standards with samples, monomeric stocks of Aβ1–40 and Aβ1–42 were prepared in Tris-buffered saline, pH 7.4 containing 0.5 M GuHCl, 0.05% Tween 20 and 1% Blocker A. Assays were performed using the Meso Scale Discovery (MSD) platform and reagents from Meso Scale (Rockville, MD). Samples, standards and blanks were analyzed in triplicate as described previously [14].

The oAβ assay used to measure soluble Aβ aggregates employs our aggregate-preferring mAb, 1C22, for capture (3 μg/mL) and biotinylated 3D6 (0.4 μg/mL) for detection. ADDLs as calibrant and the buffers and wash steps were the same as for the Aβx-40 and Aβx-42 assays [28].

### Immunoprecipitation/Western blot detection of Aβ

Mock-AD extract (0.5 ml) was incubated with 10 µL purified S97 antibody and 15 μL PAS beads overnight at 4 °C with gentle agitation. Aβ-antibody-PAS complexes were collected by centrifugation and washed as described [43]. Beads were eluted by boiling in 15 μL of 2 × sample buffer (50 mM Tris, 2% w/v SDS, 12% v/v glycerol with 0.01% phenol red), and samples electrophoresed on hand-cast, 15 well 16% polyacrylamide tris-tricine gels. Proteins were transferred to 0.2 µm nitrocellulose at 400 mA and 4 °C for 2 h. Blots were microwaved in PBS and Aβ was detected using anti-Aβ monoclonal antibodies 2G3 and 21F12, and bands visualized using a Li-COR Odyssey infrared imaging system (Li-COR, Lincoln, NE). Synthetic Aβ1-42 was loaded to allow comparison between gels.

### Production of humanized IgGs

Avastin was obtained from Myoderm Inc. (Norristown PA). Antibody sequences for aducanumab (Adu), BAN2401 (BAN), bapinezumab (Bapi), gantenerumab (Gant) and SAR228810 (SAR) were based on those from the patent literature. The variable domain sequences of 1C22 (Table 1) were derived from our 1C22 murine hybridoma using standard PCR methods. The in-house antibody, 1C22, is not in clinical development and has been described previously [19]. Synthetic DNA constructs were cloned into pTT5 expression vectors and recombinant IgGs produced in 293Expi cells (Thermo Fisher Scientific, Waltham MA). Culture supernatants were harvested and antibodies purified using mAb Select Sure resin (GE Healthcare Life Sciences, Waltham, MA) and size exclusion chromatography (SEC). Antibodies were exchanged into 10 mM Histidine buffer pH 6.0 containing 8% sucrose and stored as stocks of 1 mg/mL at − 80 °C. mAbs were tested for their ability to bind aggregated and monomeric synthetic Aβ. All anti-Aβ mAbs bound avidly to synthetic Aβ fibrils but varied in their ability to bind Aβ monomer (Additional files 2 and 3: Figs. S2 and S3). In agreement with prior studies, Bapi bound monomers most tightly and Adu bound monomer to a lesser extent than the other mAbs [1, 3, 10, 19, 35].

### Enzyme-linked immunosorbent assay (ELISA) binding assay

Microtiter plates were coated with 2.5 µg/mL of anti-Aβ antibody 4G8 (Biolegend, San Diego, CA) overnight at 4 °C. Plates were blocked with BSA then incubated with Aβ at 1 µg/mL in 1% BSA in Tris-buffered saline, pH 7.4 containing 0.05% Tween 20 (BSA/TBST) for 1 h at room temperature. Serial dilutions of recombinant anti-Aβ (hIgG1) and control (Ava) antibodies were prepared in BSA/TBST. Detection of bound antibodies was achieved with HRP-goat-anti-hIgG(H + L) (Thermo Fisher Scientific, Waltham, MA) and 3,3′,5,5′-tetramethylbenzidine TMB substrate (Thermo Fisher Scientific, Waltham, MA) on an EnVision-PE plate reader (450 nm). Binding curves and EC_50_ values were calculated using non-linear regression (four parameters) analysis of log versus response in Prism Graphpad (La Jolla, CA). Values reported are the average of triplicates and representative of 3 separate experiments.

### Surface plasma resonance (SPR) affinity determination

Analysis was performed on a Biacore T100 with HBS-EP + (10 mM HEPES, 150 mM sodium chloride, 3 mM EDTA and 0.005% P20) as the running buffer. Series S Protein A sensor chips (GE Healthcare Life sciences, Marlborough, MA) were used for the analysis. Antibodies were diluted to 5 µg/mL in HBS-EP+. SEC-purified Aβ1–42 monomer was diluted in HBS-EP + to produce a 1000 nM (4.5 µg/mL) stock, which was then serially diluted two-fold to 500, 250, 125, 62.5, 31.25 and 15.6 nM. Stock solution of Aβ1–42 protofibrils at 100 nM (66 µg/mL) was diluted in HBS-EP+ to yield a dilution series from 50 to 1.56 nM. Antibodies were flowed over the Protein A chip at 10 µL/min for 60 s, and then the Aβ (monomers or protofibrils) was injected at 30 µL/min for 180 s followed by a 360 s period to allow dissociation. The Protein A surface was regenerated with 10 mM glycine–HCl, pH 1.7. The resulting sensorgrams were double-referenced and fit to a 1:1 binding model to determine K_a_, K_d_ and K_D_.

### Mice

Wild type (WT) C57BL/6 mice were purchased from Jackson Labs (Bar Harbor, ME) and bred in-house. Animals were group housed (3–5 mice/cage) in a room with a 12 h light and 12 h dark cycle (lights on 7:00 a.m.) with ad libitum access to food and water. All animal procedures were performed in accordance with the National Institutes of Health Policy on the Use of Animals in Research and were approved by the Harvard Medical School Standing Committee on Animals and Brigham and Women’s Hospital’s Institutional Animal Care and Use Committee.

### Brain slice preparation

Slices were prepared from 2–3 months old mice as described previously [50, 51]. Briefly, animals were anaesthetized with isoflurane, decapitated, and brains were rapidly removed and immediately immersed in ice-cold (0–4 °C) artificial cerebrospinal fluid (aCSF). The aCSF contained (in mM): 124 NaCl, 3 KCl, 2.4 CaCl_2_, 2 MgSO_4_·7H_2_O, 1.25 NaH_2_PO_4_, 26 NaHCO_3_ and 10 D-glucose, and was equilibrated with 95% O_2_ and 5% CO_2_, pH 7.4, 310 mOsm. Coronal hippocampal brain slices (350 µm) [51] were cut using a Leica VT1000 S vibratome (Leica Biosystems Inc, Buffalo Grove, IL), and transferred to an interface chamber in aCSF and incubated first at 34 ± 5 °C for 30 min and then at room temperature for 1 h before recording. Slices were viable for recording for at least 4 h.

### Long-term potentiation (LTP) recording

Brain slices were transferred to a submerged recording chamber (26 ± 5 °C) superfused (10 mL/min) with oxygenated (95% O_2_ and 5% CO_2_) aCSF 20 min before electrophysiological recordings. Brain slices were visualized using an infrared and differential interference contrast camera (IR-DIC camera, Hitachi, Japan) mounted on an upright Olympus microscope (BX50WI, Olympus, Tokyo, Japan) and upright Zeiss microscope (Axio Examerner.A1, Thornwood, NY). Recording electrodes were pulled from borosilicate glass capillaries (Sutter Instruments, Novato, CA) using a micropipette puller (Model P-97; Sutter Instruments, Novato, CA) with resistance ~ 2 MΩ when filled with aCSF. To induce field excitatory post-synaptic potentials (fEPSPs) in the striatum radiatum of hippocampal CA1, a tungsten wire stimulating electrode (150 µm in diameter, FHC, Inc., Bowdoin, ME) was placed on the Schaffer collaterals of the CA3, and a recording electrode was placed at least 300 µm away on the striatum radiatum of the CA1. Test stimuli were delivered once every 20 s (0.05 Hz), and the stimulus intensity was adjusted to produce a baseline fEPSP of 30–40% of the maximal response of the initial slope of fEPSP. Thirty min following addition of sample, LTP was induced by theta burst stimulation (TBS). TBS involved 3 trains, each of 4 pulses delivered at 100 Hz, 10 times, with an interburst interval of 200 ms with a 20 s interval between trains. Field potentials were recorded using a Multiclamp amplifier (Multiclamp 700B; Molecular Devices, Sunnyvale, CA) coupled to a Digidata 1440A digitizer. Signal was sampled at 10 kHz and filtered at 2 kHz and data were analyzed offline using Clampex 10 software (Molecular Devices, Sunnyvale, CA).

### Sample application on LTP experiments

Samples were allowed to thaw at room temperature and then gently vortex mixed. When appropriate, antibodies were added to brain extracts and gently shaken (STR6, Stnart Scientific, Staffordshire, UK) for 30 min prior to addition to slices. After a stable baseline had been achieved for at least 10 min, samples were added to the aCSF reservoir. The total volume of the perfusion system was 9.5 mL, such that the effective dilution of each sample was 1:20. The experimenter was blinded to the identity of brain extracts, antibodies and aCSF vehicle. Treatments were tested in an interleaved manner to avoid variances in mice or slice quality that could influence results. Slices in each group came from different animals unless otherwise noted.

### iPSC-derived human neurons (iNs)

**Fig. 1:**
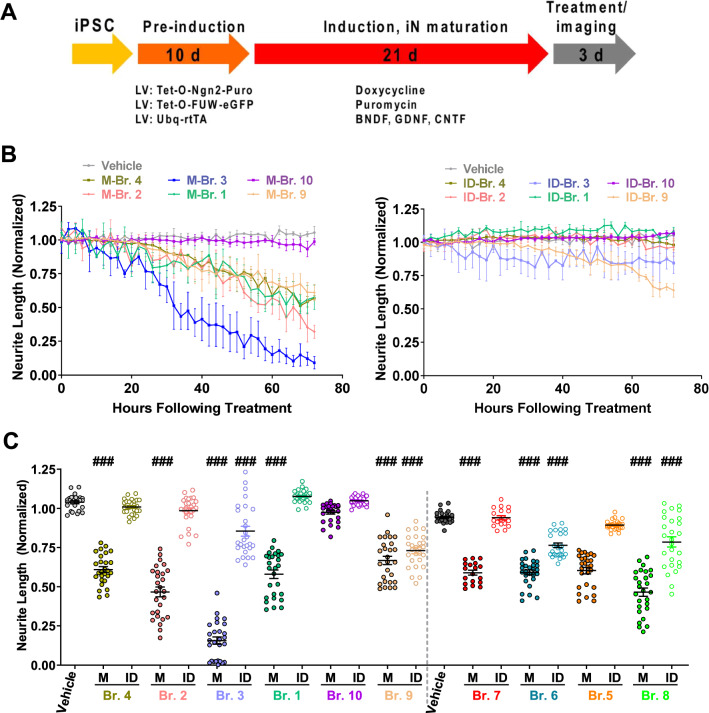
Aqueous extracts of only certain AD brains are neuritotoxic. **A** Schematic depicts the process used to generate iPSC-derived human neurons (iNs), and the timing of sample addition and live cell imaging. **B** iNs were treated with medium, mock-immunodepleted (Mock ID) AD brain extracts (left panel), or extracts immunodepleted of Aβ with antiserum S97 (ID, right panel). Each well of iNs was imaged for 6 h prior to addition of sample and NeuroTrack-identified neurite length calculated. Mock-ID and ID were tested at 1:4 dilution and cells treated with medium alone were used to monitor the integrity of untreated cells. Values are the average of triplicate wells ± SEM. **C** Plots of neurite length normalized to 6 h pre-treatment values are shown for each of 3 wells for the last 9 time points (M: mock ID; ID: S97 ID), i.e., a total of 27 data points per treatment. Compared with medium alone, mocked-ID extracts Br.4, Br.2, Br.1, Br.7, and Br.5 induced significant neuritotoxicty (Br.4, *p* < 0.0001; Br.2, *p* < 0.0001; Br.1, *p* < 0.0001; Br.7, *p* < 0.0001, and Br.5, *p* < 0.0001; One-way ANOVA test); whereas the same extracts immunodepleted of Aβ were not different from the medium control (ID-Br.4, *p* = 0.93; ID-Br.2, *p* = 0.37; ID-Br.1, *p* = 0.79; ID-Br.7, *p* = 0.99; and ID-Br.5, *p* = 0.29; One-way ANOVA test). Neither mock-ID extract of Br.10 nor it's ID-extracts (ID-Br.10) evinced neuritotoxicity during the 3-day treatment (Br.10, *p* = 0.18 and ID-Br.10, *p* = 0.99; One-way ANOVA test). However, both the mock and S97 ID extracts of Br.3, Br.9, Br.6, and Br.8 caused neurites retraction (Br.3, *p* < 0.0001; ID-Br.3, *p* < 0.0001; Br.9, *p* < 0.0001; ID-Br.9, *p* < 0.0001; Br.6, *p* < 0.0001; ID-Br.6, *p* < 0.0001; Br.8, *p* < 0.0001, and ID-Br.8, *p* < 0.0001; One-way ANOVA test). Mean values ± SEM are derived from triplicate wells merged at 2 h intervals between 56–72 h. ###*p* < 0.0001. In addition, pairwise analysis demonstrated statistically significant differences between each of the Mock and ID samples. Mock Br.4 vs. ID Br.4 *p* < 0.0001; Mock Br.2 vs. ID Br.2 *p* < 0.0001; Mock Br.3 vs. ID Br.3 *p* < 0.0001; Mock Br.1 vs. ID Br.1 *p* < 0.0001; Mock Br.10 vs. ID Br.10 *p* < 0.0001; Mock Br.9 vs. ID Br.9 *p* = 0.085; Mock Br.7 vs. ID Br.7 *p* < 0.0001; Mock Br.6 vs. ID Br.6 *p* < 0.0001; Mock Br.5 vs. ID Br.5 *p* < 0.0001; Mock Br.8 vs. ID Br.8 *p* < 0.0001 (Two-tailed paired t-test)

Neurogenin 2 (Ngn2)-induced human neurons [59] were prepared as summarized in Fig. 1A and as described previously [14, 19, 23]. Briefly, YZ1 iPSCs were maintained in media containing DMEM/F12, Knockout Serum Replacement, pencillin/streptomycin/glutamine, MEM-NEAA, and 2-mercaptoethanol (all from Invitrogen, Carlsbad, CA) plus 10 μg/mL bFGF (Millipore, Billerica, MA). iPSCs were then plated at a density of 95,000 cells/cm^2^ for viral infection at the following concentrations: pTet-O-NGN2-puro: 0.1 µL/50,000 cells; Tet-O-FUW-eGFP: 0.05 µL/50,000 cells; Fudelta GW-rtTA: 0.11 µL/50,000 cells (Alstem, Richmond, CA). To induce Neurogenin 2 expression, doxycycline was added on “iN day 1” at a concentration of 2 µg/mL, and puromycin was added on iN day 2 at 10 mg/mL and maintained in the media at all times thereafter. On iN day 4, cells were plated at 5000 cells/well on Matrigel (BD Biosciences, San Jose, CA) coated Greiner 96 well microclear plates and maintained in media consisting of Neurobasal medium (Gibco), Glutamax, 20% Dextrose, MEM-NEAA and B27 with BDNF, CNTF, GDNF (PeproTech, Rocky Hill, NJ) each at a concentration of 10 ng/mL. The neurite number and expression of neural markers reached maximal levels by iN day 14 and by iN day 21 spontaneous firing of the iNs had plateaued [19, 23]. To investigate the effects of AD brain extracts on neuritic integrity, cells were used at iN day 21.

### Sample addition to iNs and live-cell imaging

At post-induction day 21, neurons were used to investigate the effects of samples on neuritic integrity. About 7 h prior to adding the sample, images were collected from 4 fields per well every 2 h for a total of 6 h, and baseline neurite length and branch points were calculated. During this time, samples were exchanged into neurobasal medium supplemented with B27/Glutamax using PD MidiTrap G-25 columns (GE Healthcare Life Science, Milwaukee, WI). Following the 6 h baseline imaging, half of the medium was removed from each well (leaving ~ 100 µL) and 50 µL of exchanged extract or vehicle were added along with 50 µL of fresh medium. Thereafter, images were collected from 4 fields/well every 2 h for at least 72 h (Additional file 1: Fig. S5A). Phase contrast image sets were analyzed using IncuCyte Zoom 2016A Software (Essen Bioscience, Ann Arbor, MI). The analysis job Neural Track was used to automatically define neurite processes and cell bodies based on phase contrast images. Typical settings were: Segmentation Mode—Brightness; Segmentation Adjustment: 1.2; Cell body cluster filter: minimum 500 μm^2^; Neurite Filtering: Best; Neurite sensitivity: 0.4; Neurite Width: 2 μm. Total neurite length (in mm) and number of branch points were quantified and normalized to the average value measured during the 6 h period prior to sample addition. AD brain extracts (± immunodepletion) were added to neurons ± test mAbs. Five clinical mAbs (Adu, BAN, Bapi, Gant and SAR), a humanized in-house Aβ aggregate-specific mAb 1C22, & a non-Aβ antibody (Ava) were compared blinded to mAb identity.

### Data analysis and statistical tests

Figures showing IP/WB and MSD Aβ immunoassay data are representative of at least 3 independent experiments. For electrophysiological experiments, the brain extracts (Mock-ID and ID-AD) and aCSF samples were coded and tested in an interleaved manner to avoid variances in animals or slice quality influencing results. Slices in each group came from different mice unless otherwise noted. Electrophysiological data were analyzed offline by pclamp 10.2 (Molecular Devices, Sunnyvale, CA) and tested with one-way or two-way analysis of variance (ANOVA) with Bonferroni post-hoc tests or Student’s t-tests. For live-cell IncuCyte imaging, samples and treatments were coded and tested blindly. Differences between groups were tested with two-way analysis of variance (ANOVA) with Bonferroni post-hoc tests or Student’s *t* tests. #*p* < 0.05, ##*p* < 0.01, and ###*p* < 0.001. Were appropriate, pairwise analysis was also conducted.

## Results

We recently combined the use of Aβ-rich aqueous extracts of AD brain and live-cell imaging of iPSC-derived human neurons (iNs) to quantify the relative protective effects of three different anti-Aβ antibodies on Aβ-induced neuritic dystrophy [19]. Here, we applied this paradigm to address three new issues: (1) to ascertain whether the neuritotoxic activity of brain extracts in our live-cell imaging platform could predict disruption of hippocampal synaptic plasticity; (2) to examine whether well-defined biochemical measures of Aβ species relate to their bioactivity; and (3) to quantify the relative protective abilities of 5 humanized anti-Aβ monoclonal antibodies that are currently or have recently been in human trials.

### Water-soluble extracts of AD brains which exhibit Aβ-dependent neuritotoxicity also impair hippocampal LTP

**Fig. 2:**
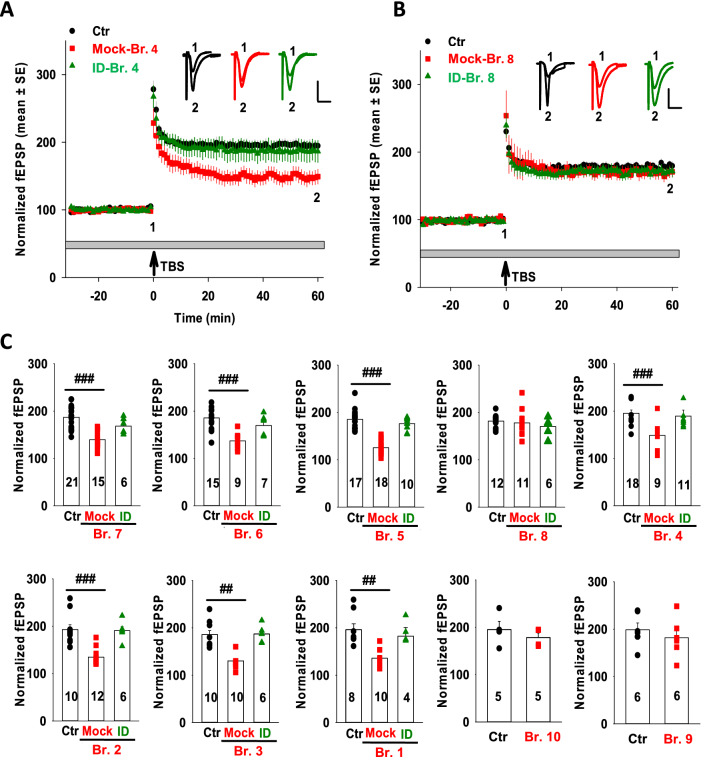
Aqueous extracts that block LTP do so in an Aβ-dependent manner. **A** Time course plots show that the aqueous extract of AD brain Br.4 (mock-Br.4) blocked hippocampal LTP, whereas the same extract which had been depleted of Aβ (ID-Br.4) did not. aCSF control is shown in black circles, mock-Br.4 in red squares, and ID-Br.4 in dark green triangles. Each slice used for each treatment was from a different animal. The gray horizontal bar indicates the period when sample was present in the bath. 1, 2, indicate example traces from time points just prior to the theta burst stimulation (↑ TBS) (1) and 60 min after TBS (2), respectively. Scale bars: 0.5 mV, 10 ms. **B** Time course plots show that the aqueous extract of brain Br.8 did not block LTP. aCSF control is shown in black circles and treatment with Br.8 extract in red squares. The gray horizontal bar indicates the period when sample was present in the bath. 1, 2, indicate example traces from time points just prior to the theta burst stimulation (↑ TBS) (1) and 60 min after TBS (2), respectively. Each slice used for each treatment was from a different animal. Scale bars: 0.5 mV, 10 ms. **C** Histogram plots of the average potentiation for the last 10 min of recording show that 7 of 10 brain extracts block LTP. Aqueous extracts from Br.7 (n = 15 vs. Ctr n = 21: F = 4.13, *p* = 1.37E-7), Br.6 (n = 10 vs. Ctr n = 14: F = 4.3, *p* = 0.003), Br.5 (n = 18 vs. Ctr n = 17: F = 4.14, *p* = 8.43E-12), Br.4 (n = 9 vs. Ctr n = 18: F = 4.24, *p* = 0.0001), Br.2 (n = 12 vs. Ctr n = 10: F = 4.35, *p* = 2.83E-5), Br.3 (n = 8 vs. Ctr n = 8: F = 4.6, *p* = 0.0002) and Br.1 (n = 8 vs. Ctr n = 8: F = 4.6, *p* = 0.0007) blocked LTP; whereas the same samples immunodepleted of Aβ did not affect LTP relative to the vehicle control (ID-Br.7, n = 6 vs. Ctr n = 21; F = 4.24, *p* = 0.08; ID-Br.6, n = 7 vs. Ctr n = 14: F = 4.38, *p* = 0.23; ID-Br.5 n = 10 vs. Ctr n = 17: F = 4.24, *p* = 0.24; ID-Br.4 n = 9 vs. Ctr n = 18: F = 4.21, *p* = 0.66; ID-Br.2 n = 5 vs. Ctr n = 10: F = 4.75, *p* = 0.66; ID-Br.3: n = 6 vs. Ctr n = 8: F = 4.75, *p* = 0.94; and ID-Br.1: n = 4 vs. Ctr n = 8: F = 4.95, *p* = 0.83; One Way ANOVA test). Extracts of Br.8 (n = 11 vs. Ctr n = 12: F = 4.32, *p* = 0.67), Br.10 (n = 5 vs. Ctr n = 5: F = 5.32, *p* = 0.27) and Br.9 (n = 6 vs. Ctr n = 6; F = 4.96, *p* = 0.47) did not block LTP. In each case, the aCSF control is shown in black circles; treatment with AD brain extracts in red squares and Aβ depleted extracts (ID) in dark green upward triangles. One Way ANOVA test; ##*p* < 0.001, ###*p* < 0.0001

We prepared aCSF extracts from the brains of 10 humans who died with mild/moderate AD (Table 2) and tested each for disease relevant bioactivity. First, we assessed whether extracts altered the neuritic architecture of human neurons (Fig. 1 and Additional file 4: Fig. S4), and then we examined whether the same extracts could alter LTP in mouse hippocampal slices (Fig. 2). In initial experiments, extracts of brains that had been treated with pre-immune serum (Mock), or immunodepleted of Aβ using the anti-Aβ polyclonal serum S97 (ID) were tested on iNs at a dilution of 1:4 (Fig. 1B and Additional file 4: Fig. S4). Compared to the vehicle control, extracts from nine of the ten AD brains caused time-dependent reductions in neurite length (Fig. 1B and Additional file 4: Fig. S4) and branch points (see e.g., Additional file 5: Fig. S5C). Prior immunodepletion of Aβ with S97 mostly or completely abrogated the neuritotoxicity of eight of these nine brain extracts (Br.1, Br.2, Br.3, Br.4, Br.5, Br.6, Br.7, and Br.8) (Fig. 1B, C, and Additional file 4: Fig. S4). The extent of neuritotoxicity was dose-dependent such that when mock extracts were used at a dilution of 1:8, the decrease in neurite length was less than at a dilution of 1:4 (see e.g., Additional file 5: Fig. S5). Extract of brain Br.9 that had been mock-ID’d or S97 ID’d caused near identical reductions in neurite length (Fig. 1B, C), indicating that the neuritotoxicity induced by this extract was not mediated by Aβ. Extracts of brain Br.10 did not alter neurite length irrespective of whether extracts were S97- or mock-immunodepleted (Fig. 1B, C). Aβ immunodepletion significantly reduced neuritotoxicity induced by Br.6 and Br.8 but not as robustly as for other extracts (Fig. 1C and Additional file 4: Fig. S4).

Next, we assessed whether the same AD brain extracts could impair LTP in mouse hippocampal slices. Strikingly, of the 8 extracts which caused Aβ-dependent neuritotoxicity, 7 (Br.1, Br.2, Br.3, Br.5, Br.5, Br.6, Br.7) also mediated an Aβ-dependent block of LTP (Fig. 2). The one mismatch was extract Br.8. It is noteworthy that while extract Br.8 caused Aβ-dependent neuritotoxicity, the signals produced by both mock-Br.8 and ID-Br.8 were more variable than those detected for most other extracts (Fig. 1C and Additional file 4: Fig. S4). Importantly, extract Br.10 which failed to cause neuritotoxicity, also failed to block LTP, and extract Br.9 which caused neuritotoxicity but in a manner independent of Aβ did not affect LTP. This finding is consistent with our prior preliminary study which suggested that a minority of AD brain extracts perturb LTP in a manner dependent on tau 31.

In our lab we routinely assessed 4, 96 well plates of iNs in a single experiment with a data collection period of 3 days. This allows the potential assessment of 80 conditions each tested in triplicate inclusive of relevant negative and positive plate controls. Correspondingly, the total data collection time for triplicate biological experiments is 9 days. In contrast, for LTP experiments it would take at least 40 days to test 80 conditions inclusion of appropriate interleaved controls. Thus, the iN system allows a shorter data collection period, while advancing the 3R’s (Replacement, Reduction and Refinement) of animal use.

Collectively, these results suggest that our medium throughput, automated live-cell imaging platform is a useful indicator of Aβ-mediated disruption of synaptic plasticity and is suitable for use in a screening funnel prior to analyses of synaptic function.

### No clear relationship between the amounts or forms of Aβ and the extent of adverse Aβ-dependent neuroactivity

**Fig. 3:**
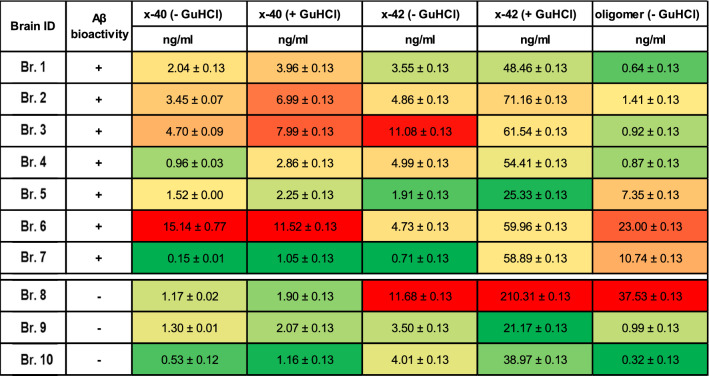
Synaptotoxic activity of brain extracts is not correlated with the measured levels of Aβ monomers nor soluble aggregates in AD. Data are grouped based on bioactivity. Active extracts are listed first, and inactive extracts second. Aβ concentrations (in ng/mL) or relative intensity are color coded to indicate the relative abundance of the different forms of Aβ. Red denotes the samples with the highest Aβ concentration and dark green is the samples with the lowest Aβ concentration. Generally, extracts from active brains contained higher levels of Aβ than those from inactive brain extracts, however, there was considerable overlap in the amounts and forms of Aβ in active and inactive extracts

Evolving data indicate that the vast bulk of Aβ present in aqueous extracts made from AD cortex is biologically inactive [14, 55]. In an effort to identify forms of Aβ which correlate with bioactivity, we quantified Aβ in extracts using IP/WB as well as three different MSD-based immunoassays (Fig. 3 and Additional file 6: Fig. S6). For IP/WBs (Additional file 6: Fig. S6A), extracts were IP’d with the same polyclonal antiserum, S97, which effectively removed bioactive Aβ from samples used in our neuritotoxicity and LTP experiments (Figs. 1, 2). In the examples shown the bulk of Aβ was captured with the first round of ID, with diminishing amounts of Aβ removed in the second and third rounds of ID (Additional file 7: Fig. S7). IP with S97 allows the capture of both native monomeric Aβ and aggregated Aβ (Additional file 1: Fig. S1). The captured Aβ was then released and denatured by boiling in SDS sample buffer, electrophoresed on SDS–polyacrylamide gels and Western blotted using mAbs specific for Aβ40 and Aβ42 [43]. This procedure revealed a similar pattern in all ten brain extracts, a broad smear centered around 7 kDa, and 2–3 bands between ~ 3.5 and 4.5 kDa (Additional file 3: Fig. S6A). In earlier studies using mass spectrometry, we demonstrated that the ~ 3.5–4.5 kDa bands constitute Aβ monomers with distinct N-termini and that the ~ 7 kDa smear contains covalently cross-linked Aβ heterodimers [4, 28]. The relative intensity of the monomer and dimer bands varied among samples (Additional file 6: Fig. S6A).

Prior work indicated that the bulk of the Aβ monomers and dimers detected on Western blots are derived from SDS-labile higher molecular weight assemblies, such that IP/WB cannot differentiate between native monomers and dimers versus monomers and dimers derived from the SDS-mediated breakdown of larger assemblies [14, 28]. Nonetheless, IP/Western blotting provides a means to assess the relative amounts of total monomer (i.e., native and aggregate-derived) versus dimer (native and aggregate-derived).

To assess native monomer content, we analyzed samples using two MSD-based immunoassays: one which preferentially recognizes Aβ40 monomers and the other Aβ42 monomers [26, 28]. Levels of Aβ40 monomers ranged from 0.53 to 15.14 ng/mL and those of Aβ42 monomers from 0.71 to 11.68 ng/mL, and most brain extracts contained substantially more Aβ42 monomer than Aβ40 monomer (Fig. 3). Most Aβ aggregates in aqueous brain extracts are labile in 5 M GuHCl [26, 28], so we used our MSD monomer assays to measure Aβ content of extracts that had been pre-incubated in GuHCl. Strikingly, treatment with GuHCl allowed detection of much higher levels of Aβ42. In all cases, the GuHCl-treated sample allowed detection of at least fivefold higher levels of Aβ42 than the corresponding untreated sample, and in eight cases the levels of GuHCl treated samples was more than ten-fold higher (Fig. 3). In contrast, GuHCl typically caused a doubling of Aβ40 levels, and for most samples the values obtained in the presence and absence of GuHCl were similar. These results indicate that aqueously soluble aggregates are largely composed of Aβ42 and that native monomers constitute only a small portion of the Aβ present in ultracentrifuged clarified aqueous brain homogenates. In an orthogonal approach, we used an MSD-based immunoassay which employs mAb 1C22 as the capture antibody and thus preferentially detects soluble Aβ aggregates; we refer to this as the oAβ assay [26, 56]. The levels of oAβ in 5 of the 10 extracts differed by less than 30% (Fig. 3). The extract from brain Br.10 contained the lowest level of soluble aggregates (0.323 ng/mL), whereas brain extract Br.8 (37.53 ng/mL) had the highest levels (Fig. 3 and Additional file 6: Fig. S6B).

To search for a relationship between the 5 different MSD-measured forms of Aβ and bioactivity, we superimposed heat map colors onto tabulated values of each Aβ analyte (Fig. 3). Importantly, we observed no simple relationship between the amounts of any form of Aβ and the extent of adverse neuroactivity (compare Figs. 1, 2, 3 and Additional file 6: Fig. S6). However, it is notable that the 2 extracts which lacked Aβ-dependent activity (Br.10 and Br.9) also tended to have the lowest levels across the 5 MSD-measured forms of Aβ and the IP/WB Aβ signal (Figs. 1, 2, 3 and Additional file 6: Fig. S6). Since MSD values for a given analytes can vary by more than two orders of magnitude between brains (Fig. 3), data in Additional file 6: Fig. S6B are normalized relative to the brain extract with the highest Aβ content for that analyte.

### Certain anti-Aβ antibodies protect against Aβ-induced neuritotoxicity by AD brain extracts more effectively than others

The results above exemplify a dilemma that has long dogged AD research, namely, how to develop and quantify efficacious anti-Aβ therapies without understanding the forms of Aβ which mediate toxicity? To address this disconnect, we used the two measures of brain-derived Aβ bioactivity described above to compare the ability of five clinically tested anti-Aβ antibodies to protect living neurons. In recent work, we reported that our aggregate-preferring anti-Aβ mAb, 1C22, effectively protected against AD brain extract-mediated neuritotoxicity [19]. This effect was dose-dependent, with 3 μg/mL of 1C22 offering near-complete protection of human neurons. Thus, our initial analyses here tested five clinical mAbs versus the humanized version of 1C22, each beginning at 3 μg/mL.

**Fig. 4:**
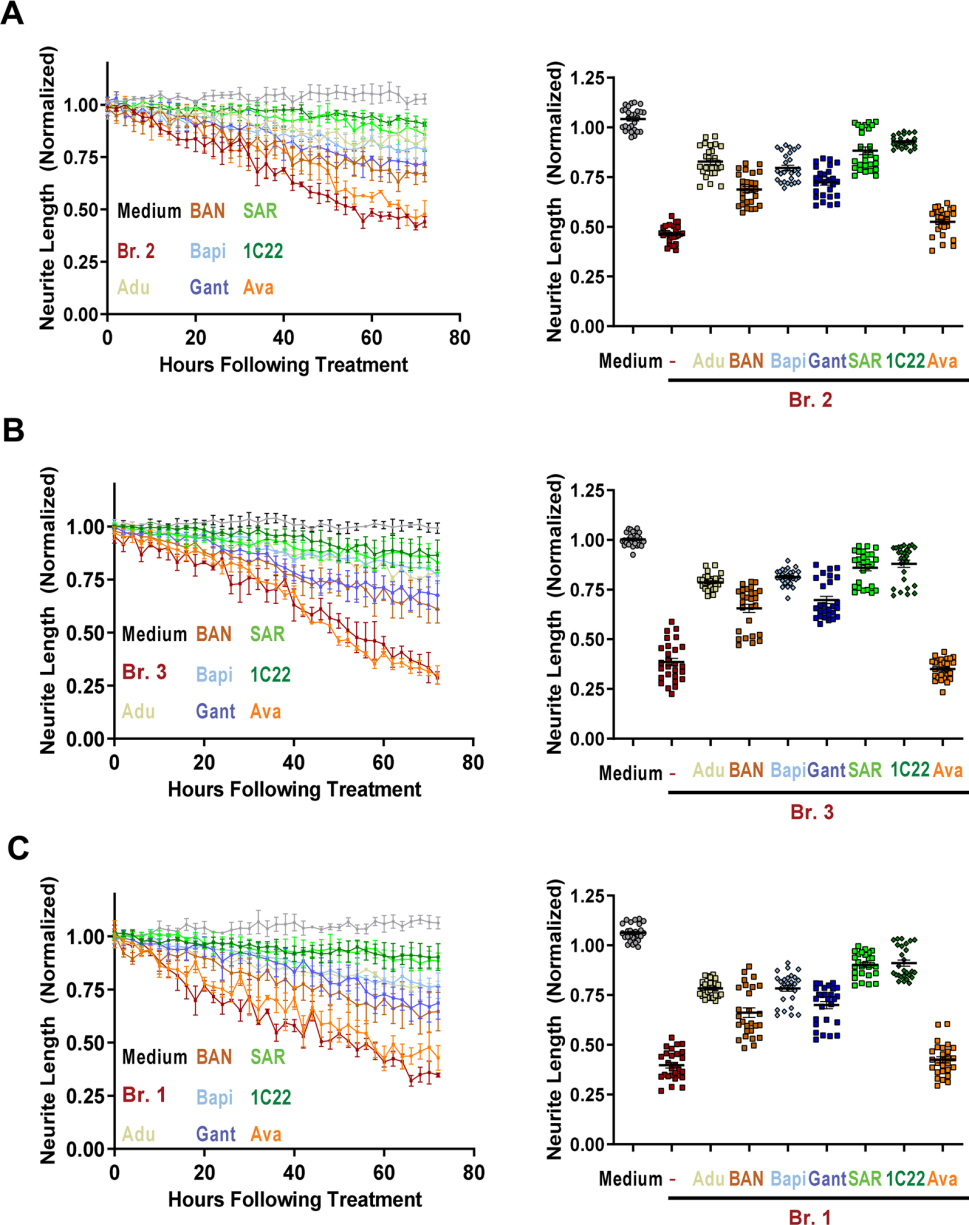
Anti-Aβ antibodies protect against neuritotoxicity induced by AD brain extracts. To determine whether clinical anti-Aβ antibodies could protect against neuritotoxicity induced by Aβ-containing AD brain extracts, iNs were treated with 1:4 diluted AD extracts Br.2 (**A**), Br.3 (**B**) or Br.1 (**C**) in the presence or absence of the indicated mAbs. Graphs on the left show time-course measurements of NeuroTrack-defined iNs neurite length. Each data point is the average of 3 wells $\pm$ SEM. Graphs on the right show plots of normalized neurite length derived from 3 wells for the last 9 time points, i.e., a total of 27 data points per treatment. Each of the six anti-Aβ mAbs, aducanumab (Adu, yellow), BAN2401 (BAN, red), bapineuzumab (Bapi, light blue), gantenerumab (Gant, dark blue), SAR228810 (SAR, light green) and 1C22 (dark green), attenuated the loss of neurites mediated by AD brain extracts, whereas the control antibody, avastin (Ava, pink), did not. In Br.2 treatment, Br.2 vs. medium, *p* < 0.0001; Br.2 + Adu vs. medium, *p* < 0.005; Br.2 + Adu vs. Br.2, *p* < 0.0001; Br.2 + BAN vs. medium, *p* < 0.0001; Br.2 + BAN vs. Br.2, *p* = 0.03; Br.2 + Bapi vs. medium, *p* < 0.0001; Br.2 + Bapi vs. Br.2, *p* < 0.0001; Br.2 + Gant vs. medium, *p* < 0.0001; Br.2 + Gant vs. Br.2, *p* = 0.0008; Br.2 + SAR vs. medium, *p* = 0.45; Br.2 + SAR vs. Br.2, *p* < 0.0001; Br.2 + IC22 vs. medium, *p* = 0.32; Br.2 + IC22 vs. Br.2, *p* < 0.0001; Br.2 + Ava vs. medium, *p* < 0.0001; Br.2 + Ava vs. Br.2, *p* = 0.97; Two-way ANOVA test. In Br.3 treatment, Br.3 alone vs. medium, *p* < 0.0001; Br.3 + Adu vs. medium, *p* < 0.0001; Br.3 + Adu vs. Br.3, *p* < 0.0001; Br.3 + BAN vs. medium, *p* < 0.0001; Br.3 + BAN vs. Br.3, *p* = 0.02; Br.3 + Bapi vs. medium, *p* < 0.0001; Br.3 + Bapi vs. Br.3, *p* < 0.0001; Br.3 + Gant vs. medium, *p* < 0.0001; Br.3 + Gant vs. Br.3, *p* = 0.0004; Br.3 + SAR vs. medium, *p* = 0.43; Br.3 + SAR vs. Br.3, *p* < 0.0001; Br.3 + IC22 vs. medium, *p* = 0.62; Br.3 + IC22 vs. Br.3, *p* < 0.0001; and Br.3 + Ava vs. medium, *p* < 0.0001; Br.3 + Ava vs. Br.3, *p* = 0.76; Two-way ANOVA test. In Br.1 treatment, Br.1 vs. medium, *p* < 0.0001; Br.1 + Adu vs. medium, *p* < 0.0001; Br.1 + Adu vs. Br.1, *p* < 0.0001; Br.1 + BAN vs. medium, *p* < 0.0001; Br.1 + BAN vs. Br.1, *p* = 0.01; Br.1 + Bapi vs. medium, *p* < 0.0001; Br.1 + Bapi vs. Br.1, *p* < 0.0001; Br.1 + Gant vs. medium, *p* < 0.0001; Br.1 + Gant vs. Br.1, *p* = 0.0005; Br.1 + SAR vs. medium, *p* = 0.06; Br.1 + SAR vs. Br.1, *p* < 0.0001; Br.1 + IC22 vs. medium, *p* = 0.15; Br.1 + IC22 vs. Br.1, *p* < 0.0001; Br.1 + Ava vs. medium, *p* < 0.0001; Br.1 + Ava vs. Br.1, *p* = 0.94; Two-way ANOVA test. Values are means ± SEM. We also calculated mean values from experiments of each mAb in A-C, to generate 3 values for each mAb from 3 experiments for multiple comparison across brain extracts. The difference for each mAb vs. unmanipulated brain extracts was highly significant (*p* < 0.0001), except for Ava (*p* = 0.9653). Compared to unmanipulated brain extracts, the mean differences are: IC22 = 0.49; SAR = 0.465; Adu = 0.383; Bapi = 0.38; Gant = 0.291; BAN = 0.252; AVA 0.03

All experiments were done blinded to the identity of the mAb being tested and included three controls, namely neurons treated with: (1) medium alone; (2) brain extract and no mAb; and (3) brain extract plus the control mAb, avastin. Avastin is a human anti-VEGF IgG1 mAb [58] which shows minimal or no reactivity towards Aβ [33]. Importantly, when tested alone at 3 µg/mL, none of the mAbs altered neuritic integrity (Additional file 8: Fig. S8). When co-administered with Br.2 extract, all six anti-Aβ mAbs afforded appreciable protection against neuritotoxicity (Fig. 4A). 1C22 allowed the highest degree of protection, with SAR, Adu and Bapi being closely similar. BAN and Gant provided slightly less protection, and avastin had no effect compared to no antibody (Fig. 4A). Closely similar results were obtained in two additional independent experiments. To establish the generalizability of these effects, we then tested all six anti-Aβ mAbs using characterized extracts from two other AD brains (Br.3 and Br.1). In each case, 1C22 and SAR performed at a high and comparable level, followed by Adu and Bapi, and then BAN and Gant (Fig. 4B, C).

**Fig. 5:**
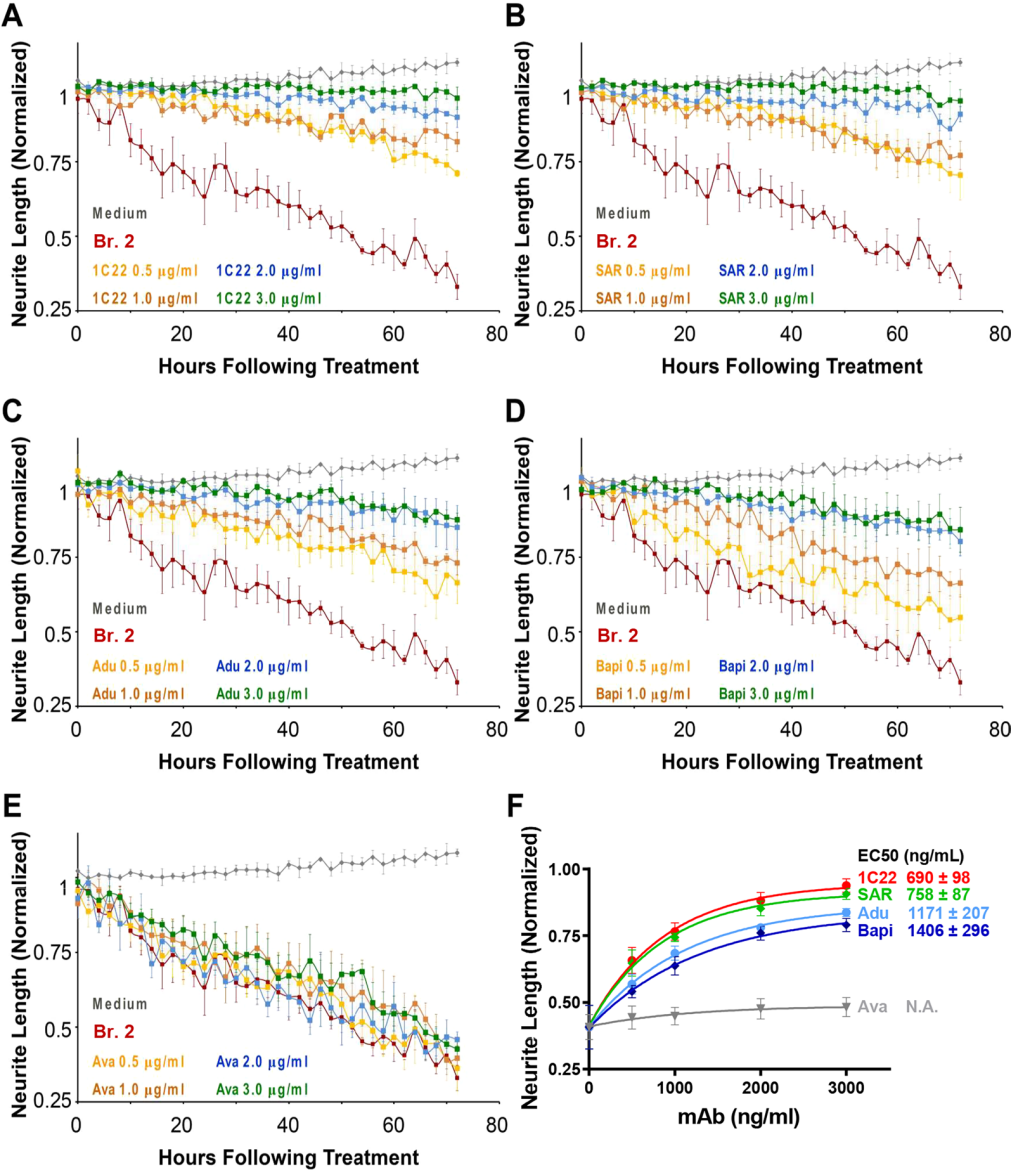
Anti-Aβ antibodies dose-dependently attenuate the neuritotoxic effects of AD brain extracts. iN day 21 cultures were treated with AD extract Br.2 at a dilution of 1:4 in the presence or absence of increasing amounts of mAbs. Graphs show time-course measurements of NeuroTrack-defined neurite length of iNs treated ± AD extract and **A** 1C22, **B** SAR228810 (SAR), **C** Aducanumab (Adu), **D** Bapineuzumab (Bapi), and **E** Avastin (Ava). Each data point is the average of 3 wells ± SEM. **F** To investigate the effect of mAb concentration, NeuroTrack-defined neurite length was averaged over the last 6 h of imaging for each treatment and values normalized to the immunodepleted AD treatment and neurite length plotted vs. antibody concentration. The half-maximal attenuation of neuritotoxicity (EC50) for 1C22, SAR, Adu and Bapi were 690 ± 98 ng/mL, 758 ± 87 ng/mL 1171 ± 207 ng/mL, and 1406 ± 296 ng/mL, respectively. The effects of mAbs, SAR performed similar to 1C22 and Adu was intermediate between SAR and Bapi (1C22 vs. SAR, no significant differences across all the tested concentrations; 1C22 vs. Adu, *p* = 0.035 at 3000 ng/mL; and 1C22 vs. Bapi, *p* = 0.036 and *p* = 0.01 at 2000 ng/ mL and 3000 ng/mL respectively. Values are the average ± SD of each condition analyzed in three separate experiments. When error bars are not visible, they are smaller than the size of the symbols. N.A.: not available

Having established that each of the six anti-Aβ mAbs could prevent neuritotoxicity, we next quantified the dose-dependent efficacy of the four best-performing mAbs (1C22, SAR, Adu and Bapi). Again, all analyses were performed blind to antibody identity. Doses of each mAb (0.5, 1.0, 2.0 and 3 μg/mL) were applied in the presence or absence of Br.2 brain extract diluted 1:4. That is, a 1:4 dilution of the original 20% (w/v) homogenate. As before, Br.2 treatment in the absence of an anti-Aβ mAb consistently reduced neurite length (Fig. 5A–E) compared to medium alone. Avastin had no protective effect, with the time-course obtained with four increasing doses overlapping that of treatment with Br.2 extract alone (Fig. 5E). Multiple comparison of Br.2 alone versus with Avastin demonstrated the quantitative reproducibility of the neuritotoxicity induced by AD-aCSF cortical extracts. All four anti-Aβ mAbs exhibited strong dose-dependent protection against Br.2-extract-induced neuritotoxicity (Fig. 5A–D; summary dose curves in F). But there were notable differences in the performance of the mAbs. In three separate experiments, 1C22 produced the most protection and Bapi the least among the four mAbs. SAR performed similar to 1C22 and Adu was intermediate between SAR and Bapi (1C22 vs. SAR, no significant differences across all the tested concentrations; 1C22 vs. Adu, *p* = 0.035 at 3000 ng/mL; and 1C22 vs. Bapi, *p* = 0.036 and *p* = 0.01 at 2000 ng/mL and 3000 ng/mL respectively; Fig. 5F). The half-maximal attenuation of neuritotoxicity (EC_50_) for 1C22, SAR, Adu and Bapi were 690 ± 98 ng/mL, 758 ± 87 ng/mL 1,171 ± 207 ng/mL, and 1,406 ± 296 ng/mL, respectively (Fig. 5F).

### Certain anti-Aβ antibodies dose-dependently protect against Aβ-mediated disruption of hippocampal synaptic plasticity

Measurement of LTP is time-consuming, relatively low-throughput and challenging to use for quantitative dose-dependent analyses of multiple mAbs. Here, we took advantage of our automated video-microscopy experiments to select two mAb concentrations to investigate the relative ability of 1C22, SAR and Adu to protect against the LTP-disrupting activity of Aβ in aqueous extracts of AD cortex. Initial experiments testing the compatibility of LTP with the mAbs alone in hippocampal slices revealed that 1C22, SAR and Adu each allowed normal LTP, whereas Bapi did not (Additional file 9: Fig. S9). Consequently, Bapi was not tested further.

**Fig. 6:**
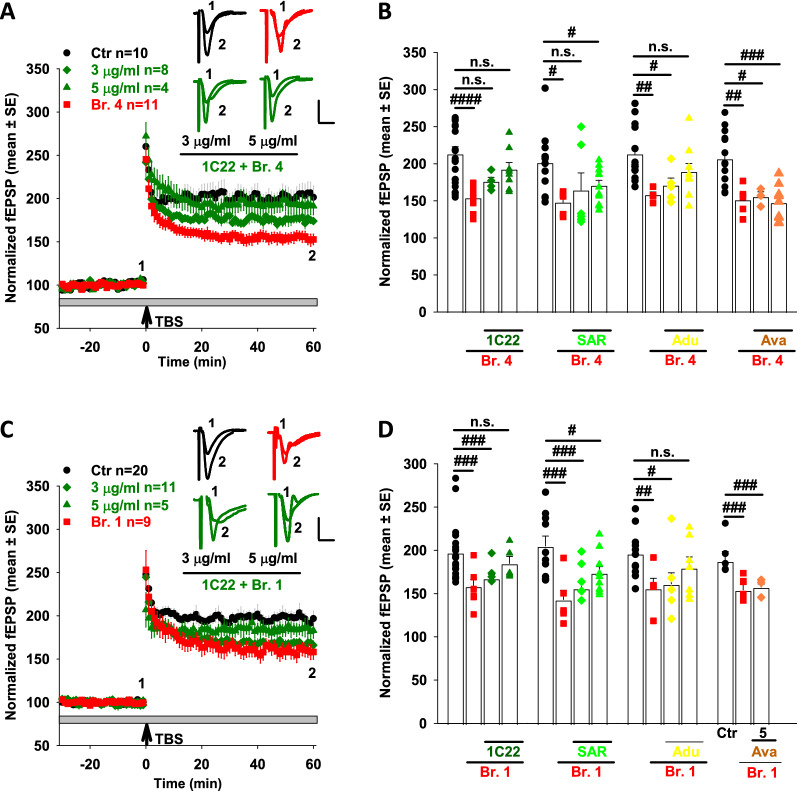
Certain anti-Aβ antibodies dose-dependently attenuate the plasticity-disrupting effects of AD brain extracts. **A** Time course plots show that 1C22 dose-dependently rescued the blockade of hippocampal LTP by AD extract Br.4. aCSF control is in black circles; Br.4 treatment is in red squares, Br.4 plus 3 µg/mL IC22 is in dark green diamonds, and Br.4 plus 5 µg/ml IC22 is in dark green upward pointing triangles. Each slice used for each treatment was from a different animal. The gray horizontal bar indicates the time period when sample was present in the bath. 1, 2, indicate example traces just prior to the theta burst stimulation (↑ TBS) (1) and 60 min after TBS (2), respectively. Scale bars: 0.5 mV, 10 ms. **B** Histogram plots of the average potentiation for the last 10 min of LTP recording for experiments testing Br.4 treatment ± 1C22, SAR, Adu and Ava at 3 and 5 µg/mL. In four separate experiments, Br.4 significantly blocked LTP compared to vehicle control (Ctr n = 15 vs. Br.4 n = 15 in 1C22 test group, F = 4.2, *p* = 1.51E-5; Ctr n = 13 vs. Br.4 n = 5 in SAR test group, F = 4.49, *p* = 0.01; Ctr n = 14 vs. Br.4 n = 4 in Adu test group, F = 4.49, *p* = 0.009; Ctr n = 15 vs. Br.4 n = 5 in Ava test group, F = 4.41, *p* = 0.003; One Way ANOVA test). All three anti-Aβ antibodies, but not control antibody (Ava), dose-dependently rescued the blockade of LTP by Br.4 (Ctr n = 15 vs. Br.4 with 3 µg/mL 1C22 n = 4, F = 4.45, *p* = 0.1; Ctr n = 15 vs. Br.4 with 5 µg/mL 1C22 n = 8, F = 4.32, *p* = 0.22; Ctr n = 13 vs. Br.4 with 3 µg/mL SAR n = 6, F = 4.45, *p* = 0.12; Ctr n = 13 vs. Br.4 with 5 µg/mL SAR n = 11, F = 4.3, *p* = 0.04; Ctr n = 14 vs. Br.4 with 3 µg/mL Adu n = 5, F = 4.45, *p* = 0.03; Ctr n = 14 vs. Br.4 with 5 µg/mL Adu n = 9, F = 4.32, *p* = 0.13; Ctr n = 15 vs. Br.4 with 3 µg/mL Ava n = 3, F = 4.49, *p* = 0.02; Ctr n = 15 vs. Br.4 with 5 µg/mL Ava n = 9, F = 4.3, *p* = 0.0001; One Way ANOVA test). In each case, aCSF control is shown in black circles; treatment with Br.4 in red squares; Br.4 plus 3 µg/mL antibodies are in diamonds, and Br.4 plus 5 µg/mL antibodies are in upward pointing triangles. **C** Time course plots show that 1C22 dose-dependently rescued the blockade of hippocampal LTP by AD extract Br.1. The aCSF control is in black circles; Br.1 treatment is in red squares, Br.1 plus 3 µg/mL IC22 is in dark green diamonds, and Br.1 plus 5 µg/mL IC22 is in dark green upward pointing triangles. Each slice used for each treatment was from a different animal. The gray horizontal bar indicates the time period when sample was present in the bath. 1, 2, indicate example traces just prior to the theta burst stimulation (↑ TBS) (1) and 60 min after TBS (2), respectively. Scale bars: 0.5 mV, 10 ms. **D** Histogram plots of the average potentiation for the last 10 min of LTP recording for experiments testing Br.1 treatment ± 1C22, SAR, Adu and Ava each at 3 and 5 µg/mL. In each of the four experiments Br.1 significantly blocked LTP compared to control (Ctr n = 20 vs. Br.1 n = 9 in 1C22 test group, F = 4.21, *p* = 0.003; Ctr n = 10 vs. Br.1 n = 6 in SAR test group, F = 4.6, *p* = 0.003; Ctr n = 11 vs. Br.1 n = 5 in Adu test group, F = 4.6 *p* = 0.02; Ctr n = 5 vs. Br.1 n = 6 in Ava testing group, F = 5.12, *p* = 0.007; One Way ANOVA test). Anti-Aβ antibodies dose-dependently attenuated the blockade of LTP by AD sample Br.1 (Ctr n = 20 vs. Br.1 with 3 µg/mL 1C22 n = 11, F = 4.18, *p* = 0.007; Ctr n = 15 vs. Br.1 with 5 µg/mL 1C22 n = 5, F = 4.28, *p* = 0.41; Ctr n = 13 vs. Br.1 with 3 µg/mL SAR n = 10, F = 4.41, *p* = 0.001; Ctr n = 13 vs. Br.1 with 5 µg/mL SAR n = 10, F = 4.41, *p* = 0.04; Ctr n = 14 vs. Br.1 with 3 µg/mL Adu n = 10, F = 4.385, *p* = 0.03; Ctr n = 14 vs. Br.1 with 5 µg/mL Adu n = 7, F = 4.49, *p* = 0.27; One Way ANOVA test). However, control antibody Ava did not rescue the LTP (Ctr n = 5 vs. Br.1 with 5 µg/mL Ava n = 5, F = 5.32, *p* = 0.009; One Way ANOVA test). In each case, aCSF control is shown in black circles; treatment with Br.1 in red squares; Br.4 plus 3 µg/mL antibodies are in diamonds, and Br.4 plus 5 µg/mL antibodies are in upward pointing triangles. 1C22 is in dark green symbols; SAR is in green symbols; Adu is in yellow symbols and Ava is in dark orange. #*p* < 0.05, ##*p* < 0.01, ###*p* < 0.001 and ####*p* < 0.0001

In a series of interleaved blinded experiments, we investigated the effect of brain extracts in the presence or absence of 3 or 5 µg/mL of 1C22, SAR or Adu (Fig. 6). mAbs were premixed with the brain extracts at room temperature for 30 min and then added to the perfusion bath and the mixture circulated over slices for 30 min prior to electrical stimulation. As expected, when administered in the absence of mAbs, extracts Br.4 and Br.1 consistently caused a block of LTP, and co-administration of Avastin did not prevent this block (Fig. 6). When tested at 3 µg/mL, IC22 and SAR rescued LTP suppression induced by Br.4, but not by Br.1 (Fig. 6B, D). Slices treated with brain extracts mixed with Adu at 3 µg/mL showed a partial but statistically insignificant recovery of LTP (Fig. 6B, D). When used at 5 µg/mL, IC22 and Adu fully rescued the suppression of LTP mediated by extracts from both AD brain extracts Br.4 and Br.1 (Fig. 6B, D). At 5 µg/mL, SAR also showed a strong trend to attenuate synaptotoxicity, but this protection did not reach statistical significance (Fig. 6B, D).

In general, the hippocampal LTP and live-neuron imaging experiments yielded similar data (compare Figs. 5, 6). All 3 mAbs dose-dependently protected against the LTP and neurite-disrupting activity of Aβ-containing brain extracts—although this effect was not always statistically significant. 1C22 was most effective at protecting against both neuritotoxicity and synaptotoxicity. Adu allowed better protection against disruption of LTP than SAR, whereas SAR produced better protection against neuritotoxicity than Adu.

## Discussion

In this study, we had three primary objectives: (1) to ascertain whether extracts with Aβ-dependent neuritotoxic activity similarly disrupted LTP; (2) to examine whether well-defined biochemical measures of Aβ species relate to their bioactivity; and (3) to quantify the relative protective abilities of five humanized anti-Aβ monoclonal antibodies that are or have recently been in human trials. Our results have provided clear answers to all three objectives.

In previous studies, we found that certain Aβ-containing human brain extracts which blocked LTP could also cause neuritotoxicity [4, 14], facilitate LTD [16, 42], disrupt E/I balance [50, 60], reduce spine density and prevent consolidation of a learned behavior [42]. However, these prior studies tested only one or two AD brain extracts and one other measure of neural activity, and no other studies included Aβ-containing AD extracts which lacked LTP-disrupting activity [4, 16, 42, 50, 60]. To address the observation that Aβ-containing extracts of different brains may exhibit different degrees of LTP inhibition [14, 42, 55], including some incapable of blocking LTP [32], we systematically tested extracts from 10 different AD brains each with varying LTP-disrupting activity for their effects on neuritic integrity.

The extent of neuritotoxic activity detected in iPSC-derived human neurons using our live-cell imaging platform was well matched to effects on LTP measured in wild-type mouse hippocampal slices. Specifically, the rank orders of these two activities across the tested brain extracts were highly similar. Two of three brain extracts which failed to show disruption of LTP also failed to induce Aβ-dependent neuritotoxicity. The third brain extract (Br.8) which did not alter LTP had a significant but highly variable effect on neuritic integrity, and immunodepletion of Aβ from this extract failed to fully protect against neuritotoxicity. These results suggest that despite significant differences in the timescales (minutes to hours for LTP, and hours to days for neuritic changes) and molecular bases of the processes, these readouts have sufficiently similar responses to neurotoxic forms of Aβ that one may be used to predict the other. Ten is a relatively large number of brain extracts, nonetheless, it would be desirable to examine extracts of yet more brains to assess the true predictiveness of these assays for each other. Recent reports indicate that it is possible to assess LTP in iPSC-derived neurons [7] and in future studies it will be interesting to compare the effects of extracts on form (neurite morphology) and function (LTP) using iNs.

Utilizing the 10 brain extracts characterized in the first part of our study, we investigated whether Aβ-dependent neuritic activity correlated with the presence and amount of certain forms of Aβ. Interestingly, measures of aggregated Aβ were the least good predictors of activity. While this observation may seem counterintuitive, it is congruent with prior reports that only a small fraction of Aβ in aqueous extracts of AD cortex is neuroactive and that activity resides in the most readily diffusible forms of Aβ [14]. These data are also consistent with the demonstration that disaggregation of large aggregates can render inactive forms of Aβ active [55] and that dimers isolated from human brain disrupt LTP and induce neuritotoxicty [4, 42]. Although we and others have developed assays capable of measuring soluble aggregates of Aβ [12, 15, 25, 39, 56], these assays detect a range of Aβ assemblies. Thus, measurement of generic soluble Aβ aggregates (*aka* oligomers) in complex biological mixtures would not be expected to discriminate between specimens with high Aβ neuroactivity and those with little or no activity. Moreover, it highly likely that other bioactive molecules (such as tau) may potentiate or attenuate Aβ activity, and that variation in the levels of such molecules could underlie differences in the Aβ-dependent bioactivity of extracts from different AD brains.

In a pilot study we examined the ability of the 6 different anti-Aβ mAbs to IP Aβ from human brain in an effort to determine: (1) if there was a discernible difference between the amount of Aβ captured by the 6 mAbs, and (2) if the mAbs detected more Aβ in an active brain extract (Br. 3) versus an inactive brain extract (Br. 9) (Additional file 10: Fig. S10). As with the other biochemical assessments there was no clear difference between brain extracts. In future, it will be important to extend the analysis of Aβ captured by different therapeutic mAbs—ideally using assays that allow assessment of native structures, and/or applying other very recently described oligomer assays (e.g., [44]).

Aducanumab, currently the sole 1approved anti-Aβ antibody, and the next two most advanced clinical antibodies, lecanemab (BAN2401) and gantenerumab, all have low affinity for Aβ monomers but bind with increasing avidity to higher order aggregates [1, 3, 10]. Using the patent-reported sequences of aducanumab, BAN2401, gantenerumab and bapineuzumab, we investigated the ability of these clinical antibodies to protect against the neuritotoxic and synaptic plasticity-disrupting effects of Aβ-containing AD brain extracts. We also included our in-house aggregate-preferring antibody 1C22 [19, 26] and SAR228810, an antibody which has completed Phase 1 in AD patients [35]. Prior to testing their bioactivity, the relative preference of all six antibodies for synthetic Aβ monomer, protofibrils and fibrils was assessed using an immunoassay and SPR. In agreement with published reports, bapineuzumab bound both monomer and fibrils with high affinity, whereas the other antibodies exhibited differing degrees of preferential recognition of fibrils and protofibrils [19, 35].

At a single fixed concentration (1.5 µg/mL), the antibodies provided varying degrees of protection against Aβ-mediated neuritotoxicity, with a rank order of 1C22 ~ SAR228810 > aducanumab ~ bapinezumab > gantenerumab > BAN2401. Notably this ranking was consistent across experiments with extracts from three different AD brains. Efficacy (EC5_50_) studies of the four most protective antibodies confirmed that 1C22 and SAR produced more effective protection than either aducanumab or bapinezumab. The robustness of the neuritotoxicty assay is demonstrated by the high degree of similarity of the EC_50s_ calculated here for 1C22 and bapineuzumab and the results obtained in an earlier study testing 1C22 and the murine precursor of bapinezumab, 3D6. Here, 1C22 and bapineuzumab had EC_50s_ of ~ 0.7 µg/mL and ~ 1.4 µg/mL, comparable to ~ 0.8 µg/mL and ~ 1.1 µg/mL in the earlier study (compare Fig. 5F to [19]—Fig. 7B). These results are all the more remarkable in that they were obtained using extracts from different AD brains, and the neurons were prepared by a different investigator.

When tested for the ability to protect against the LTP-disrupting activity of Aβ-containing brain extracts, 1C22 was again the most effective antibody examined. Just as the extent of neuritotoxic activity detected by our live-cell imaging assay was well matched to the toxic effects on LTP across extracts from 10 different AD brains, the relative protective effects of the antibodies were consistent between the two assays. Because of its higher throughput, measurement of neuritotoxicity allowed greater concentration-range exploration and therefore a finer assessment of antibody potencies (EC_50s_). An additional advantage of our neuritotoxicity assay lies in the use of well-differentiated iPSC-derived human neurons which can be genetically manipulated (e.g., using CRISPR-based approaches) to interrogate mechanisms underlying Aβ toxicity. In both assays we employed the most disease relevant source of Aβ—extracts of sporadic AD brains. This is particularly important, as aqueous extracts of AD brain contain highly heterogenous primary sequences of Aβ peptides and a mixture of Aβ aggregation states [4, 14, 28]. For instance, we detected 22 different Aβ sequences in an aqueous extract from a single brain—these included different N- and C-termini and post-translational modification such as pyroglutamylation at residues 3 and 11, and oxidation of methionine 35 [4]. Separately, we have documented a broad distribution of soluble aggregates ~ 7-kDa Aβ species up to ~ 700 kDa [28]. We have previously shown that brain extracts such as those used in the current study contain truncated and post-translationally modified Aβ [4].

The use of this natural cocktail effectively tests whether neutralizing antibodies are capable of engaging disease-relevant sequences and to what extent they are lost on inactive aggregates of brain Aβ. While none of the antibodies tested are specific for truncated or modified Aβ, all would be expected to detect a range of post-translationally modified Aβ species. It is known that both BAN and Adu can recognize certain truncated forms of Aβ [1, 18]. Of the mAbs tested, only Bapi requires the N-terminal Asp1, but even this mAb would be expected to recognize Aβ1-x with PTMs outside of its 1–5 epitope.

Since completion of our experiments a phase 2 trial of an antibody specific for N-terminal pyro-glutamate-3 Aβ (pE3-Aβ), donanemab, has yielded encouraging results in patients with early Alzheimer’s disease [30]. While pE3-Aβ is believed to form after the deposition of amyloid plaques [8] it is expected that the equilibrium between insoluble and soluble Aβ will give rise to a portion of oligomers which contain pE3. In future studies it will be important to investigate if pE3 specific antibodies can ameliorate Aβ toxicity, and how well they compare to antibodies capable of detecting both pE3 positive and pE3 negative assemblies.

We are mindful that there are many factors which determine the usefulness of an anti-Aβ antibody therapeutic. These include, but are not limited to, pharmacokinetics, the ability to productively engage microglia, and risk of producing amyloid-related imaging abnormalities (ARIA). It is unknown whether the effects of Aβ on in vitro systems are directly relevant to the in vivo human brain, and no single in vitro or in vivo assay can provide insight to the manifold factors that determine the therapeutic index and risk–benefit profile for a given immunotherapeutic in humans. Nevertheless, optimizing the ability of candidate antibodies to recognize the most bioactive human Aβ species should be central to identifying new generations of anti-Aβ antibodies that will follow the current crop of Aβ-neutralizing and amyloid-clearing antibodies. We believe that the combination of live-cell imaging of human neurons with human (AD) brain extracts should prove highly useful for optimizing the choice of future Aβ immunotherapeutics as well as other novel anti-Aβ modalities.

## Supplementary Information

**Additional file 1: Fig. S1.** Characterization of the novel anti-Aβ antiserum S97.Three rabbits (S93, S94 and S97) were repetitively immunized with aggregated synthetic Aβ1-42 and blood collected 1 week after each immunization. After the 4th immunization, all 3 rabbits produced detectable levels of anti-Aβ antibodies. Data shown are for sera collected after 7th immunization. (A) Aβ1-40 or (B) Aβ1-42 were immobilized on ELISA plates at 30 ng/well and serial dilutions of each antiserum or normal rabbit serum (NRS) added to the plate and detected with horseradish peroxidase (HRP)-conjugated secondary antibody. At dilutions in excess of 1 in a 100,000, only S97 still detected synthetic Aβ. (C) Using our well-established IP/WB protocol, the same 3 antisera were tested for their ability to immunoprecipitate Aβ species from the conditioned medium of 7PA2 CHO cells [53]. Samples were immunoprecipitated using S93, S94, S97 and NRS and Western blotted with 6E10. Immunoreactive Aβ-specific bands migrating at ~4 and ~8-10 kDa are indicated with arrows. Non-specific bands detected in samples exposed to NRS are indicated. S97 readily immunoprecipitated a range of Aβ species from 7PA2 conditioned media consistent with the pattern seen with other high affinity anti-Aβ polyclonal antibodies [43]. (D) SEC-isolated synthetic Aβ1-40 monomer and aggregated synthetic Aβ1-42 were dotted onto 0.2 μm nitrocellulose at the concentrations shown and detected with either S97 or NRS. S97 readily detected both monomeric and aggregated Aβ.

**Additional file 2: Fig. S2.** Most anti-Aβ antibodies preferentially recognize Aβ fibrils vs. monomers. (A) Plates were coated with 2.5 µg/mL of anti-Aβ antibody 4G8, Aβ samples applied, and test mAbs serially diluted across plates. Aducanumab (Adu, yellow), BAN2401 (BAN, red), bapineuzumab (Bapi, light blue), gantenerumab (Gant, dark blue), SAR228810 (SAR, light green) and 1C22 (dark green). OD values are normalized relative to Bapi, which was included in each plate. (B) Antibody binding EC50 values were calculated using a four-parameter, non-linear regression analysis of log concentration versus normalized OD.

**Additional file 3: Fig. S3.** Surface plasmon resonance reveals that all anti-Aβ antibodies preferentially recognize soluble aggregates over monomers. (A) Anti-Aβ antibodies were captured onto Protein A sensor chips and Aβ protofibrils or monomer flowed over the chip and binding assessed. (B) Kinetics data for anti-Aβ antibodies binding to protofibrils (PFs) and Aβ1-42 monomer (Mon). ND indicates that reliable estimates could not be determined.

**Additional file 4: Fig. S4.** Aqueous extracts of most AD brains are neuritotoxic. This Figure is an extension of Figure 1B and shows results for brain extracts Br.7, Br.6, Br.5 and Br.8. (A) iNs were treated with medium, mock-immunodepleted (Mock ID) AD brain extracts, or (B) extracts immunodepleted of Aβ with S97 (ID, right panel). Each well of iNs was imaged for 6 hours prior to addition of sample and NeuroTrack-identified neurite length calculated. Mock-ID and ID were tested at 1:4 dilution and cells treated with medium alone were used to monitor the integrity of untreated cells. Values are the average of triplicate wells ± SEM.

**Additional file 5: Fig. S5.** Bioactive AD brain extracts dose-dependently induce neuritotoxicity. Live-cell imaging was used to monitor the effect of an Aβ-containing AD brain extracts on iNs. (A) iN day 21 cultures were treated with medium, or mock-immunodepleted (Mock ID) BR.2 or BR.2 extract immunodepleted of Aβ with the pan anti-Aβ antiserum S97 (S97 ID) and cells were imaged for 72 hours. Phase contrast images (top panels) at 0, and 72 hours were analyzed using the IncuCutye NeuroTrack algorithm to identify neurites (middle panels), and the NeuroTrack-identified neurites (pink) are shown superimposed on the phase contrast image (bottom panels). Scale bars are 100 μm. (B) Time-course plots of neurite length (left panel) and branch points (right panel) of treatments as in A. Each well of iNs was imaged for 6 hours prior to addition of sample and NeuroTrack-identified neurite length and branch points determined and used to calculate normalized neurite length and branch points measured at each interval. Mock-ID extract BR.2 was tested at 2 dilutions, 1:4, and 1:8. ID-BR.2 was tested at 1:4 and cells treated with medium alone were used to monitor the integrity of untreated cells. Data points are the average of triplicate wells ± SEM. (C) Plots of normalized neurite length (left panel) and neurite branch points (right panel) for the last 9 time points are shown as mean values ± SEM; i.e., a total of 27 data points per treatment. Brain extracts Br.2, Br.3 and Br.1 caused dose-dependent neuritotoxicity, whereas the same extracts immunodepleted of Aβ had no effect. For neurite length measurement, ID-Br.2 vs. medium, p=0.87, Br.2 1:4 vs. medium, p<0.0001, Br.2 1:8 vs. medium, p<0.0001; ID-Br.3 vs. medium, p=1, Br.3 1:4 vs. medium, p<0.0001, Br.3 1:8 vs. medium, p<0.0001; ID-Br.1 vs. medium, p=0.93, Br.1 1:4 vs. medium, p<0.0001, Br.1 1:8 vs. medium, p<0.0001 (Two-way ANOVA test). For branch points measurement, ID-Br.2 vs. medium, p = 1, Br.2 1:4 vs. medium, p<0.0001, Br.2 1:8 vs. medium, p<0.0001; ID-Br.3 vs. medium, p=0.93, Br.3 1:4 vs. medium, p<0.0001, Br.3 1:8 vs. medium, p<0.0001; ID-Br.1 vs. medium, p=1, Br.1 1:4 vs. medium, p<0.0001, Br.1 1:8 vs. medium, p<0.0001 (Two-Way ANOVA test).

**Additional file 6: Fig. S6.** The water-soluble extracts of AD brains contain a mixture of Aβ monomers and soluble aggregates. Brain extracts were analyzed using 6 distinct approaches. (A) Samples were immunoprecipitated using S97 or preimmune serum (PI) and western blotted with combination of 2G3 (to Aβ40) and 21F12 (to Aβ42). Immunoreactive Aβ-specific bands migrating at ~4 and ~7 kDa are indicated with arrows. Non-specific bands detected in samples treated with PI are indicated. (B) Aβ monomer levels were measured using MSD-immunoassays that recognize Aβ40 and Aβ42. An assay that preferentially detects soluble aggregates (oAβ) was used to measure soluble aggregates in their native state. In an orthogonal approach, samples were pre-treated with 5 M GuHCl to disassemble soluble aggregates and the resulting Aβ40 and Aβ42 monomers were detected using the MSD Aβ40 and Aβ42 immunoassays. For IP/WB, samples were analyzed in duplicate, whereas samples were analyzed in triplicate for MSD-immunoassays. Values for MSD immunoassays are normalized to the brain extract which contained the highest amount of Aβ for a given analyte. The results shown are representative of 3 independent experiments. The raw data used to generate the graph in B are provided in Fig. 3.

**Additional file 7: Fig. S7.** The S97 pan anti-Aβ polyclonal antibody effectively depletes Aβ from AD aqueous extracts. Samples were immunoprecipitated using S97 or preimmune serum (PI) and protein A sepharose (PrA) for three rounds and an additional mop up step with PrA only. (A) Workflow of S97 immunodepetion (right panel) and mock immunodepletion (left panel). (B-D) Representative blots of IP’d materials from AD brain Br.1, Br.10 and Br.9. Aβ-specific bands were visualized with a combination of 2G3 (to Aβ40) and 12F12 (to Aβ42). Aβ monomers and dimers are indicated with single and double arrows. Non-specific bands detected in samples are indicated on the right.

**Additional file 8: Fig. S8.** Anti-Aβ mAbs alone do not alter neurite integrity. (A) iN day 21 cultures were treated with medium, or PBS (1:100 dilution) or antibody (1:100 dilution in PBS, 3 μg/mL) and cells were imaged for 72 hours. Each well of iNs was imaged for 6 hours prior to addition of sample and NeuroTrack-identified neurite length and branch points determined and used to normalize neurite length (left panel) and branch points (right panel) at each interval. The values shown in graphs are the average of triplicate wells for each treatment ± SEM. (B) Plots of normalized neurite length (left panel) and neurite branch points (right panel) are derived from 3 wells over the last 9 time points and are presented as mean values ± SEM.

**Additional file 9: Fig. S9.** Bapineuzumab but not other anti-Aβ mAbs affect LTP. (A) An example time course plots show that 1C22 alone had no effects on hippocampal basal neuronal transmission and LTP. aCSF control is shown using black circles; 3 µg/mL 1C22 treatments are shown using dark green squares; and 5 µg/mL 1C22 treatments are shown as dark green upward pointing triangles. The gray horizontal bar indicates the time period when sample was present in the bath. 1, 2, indicate example traces from time points just prior to the theta burst stimulation (↑ TBS) (1) and 60 minutes after TBS (2), respectively. Each slice used for each treatment was from a different animal. Scale bar 0.4 mV, 10 ms. (B) Histogram plots of the average potentiation for the last 10 minutes of LTP recording treated with 5 different mAbs and vehicle buffer (10 mM Histidine in 8% sucrose, pH 6.0) at 3 and 5 µg/mL. Note in order to maintain blinding of samples, 3 separate controls were tested, 2 of which are designated 3 and 5, respectively. Compared to aCSF control, vehicle buffer, 3 comparative mAbs (IC22, SAR and Adu) and control antibody (Ava) had no effects on LTP (Ctr n=15 vs. 3 µg/mL buffer n=12, F=4.3，p=0.93; Ctr n=15 vs. 5 µg/mL buffer n=8, F=4.41，p=0.72; Ctr n=12 vs. 3 µg/mL 1C22 n=7, F=4.45，p=0.67; Ctr n=15 vs. 5 µg/mL 1C22 n=5, F=4.54，p=0.55; Ctr n=12 vs. 3 µg/mL SAR n=8, F=4.41，p=0.24; Ctr n=12 vs. 5 µg/mL SAR n=6, F=4.49, p=0.53; Ctr n=8 vs. 3 µg/mL Adu n=6, F=4.75，p=0.53; Ctr n=8 vs. 5 µg/mL Adu n=6, F=4.75，p=0.61; Ctr n=7 vs. 3 µg/mL Ava n=7, F=4.75，p=0.2; Ctr n=7 vs. 5 µg/mL Ava n=5, F=4.96，p=0.17; One Way ANOVA test), whereas Bapi produced significant depression of LTP at 3 µg/mL (Bapi n=9 vs. Ctr n=11, F=4.41, p=0.005, One Way ANOVA test). aCSF control is in black circles; 3 µg/mL treatments are shown using squares and 5 µg/ml treatments with upward pointing triangles. ##p<0.01.

**Additional file 10: Fig. S10.** Anti-Aβ mAbs immunoprecipitate Aβ from AD aqueous extracts. Half milliliter aliquots of Br. 11 (A), Br.3 (B) and Br.9 (C) brain extracts were immunoprecipitated using the indicated anti-Aβ antibodies along with 10 μL of 1:1 protein A Sepharose beads:protein G agrose beads. Ava and S97 were used as negative and positive control, respectively. Aβ-specific bands were visualized using a combination of 2G3 (to Aβ40) and 12F12 (to Aβ42). The insert below each full-length blot is an image of cropped portion of the same blot, but at longer exposure. Aβ monomers and dimers are indicated with single and double arrows. Non-specific bands detected in samples are indicated on the right.

## Footnotes

1 Since acceptance of this paper, lecanemab was granted accelerated approval by the FDA.
