# Supplementary figures and images for: Learnings about Aβ from human brain recommend the use of a live-neuron bioassay for the discovery of next generation Alzheimer’s disease immunotherapeutics
Source: Acta Neuropathol Commun. 2023 Mar 10;11:39. doi: 10.1186/s40478-023-01511-2 (PMC10007750; doi:10.1186/s40478-023-01511-2)

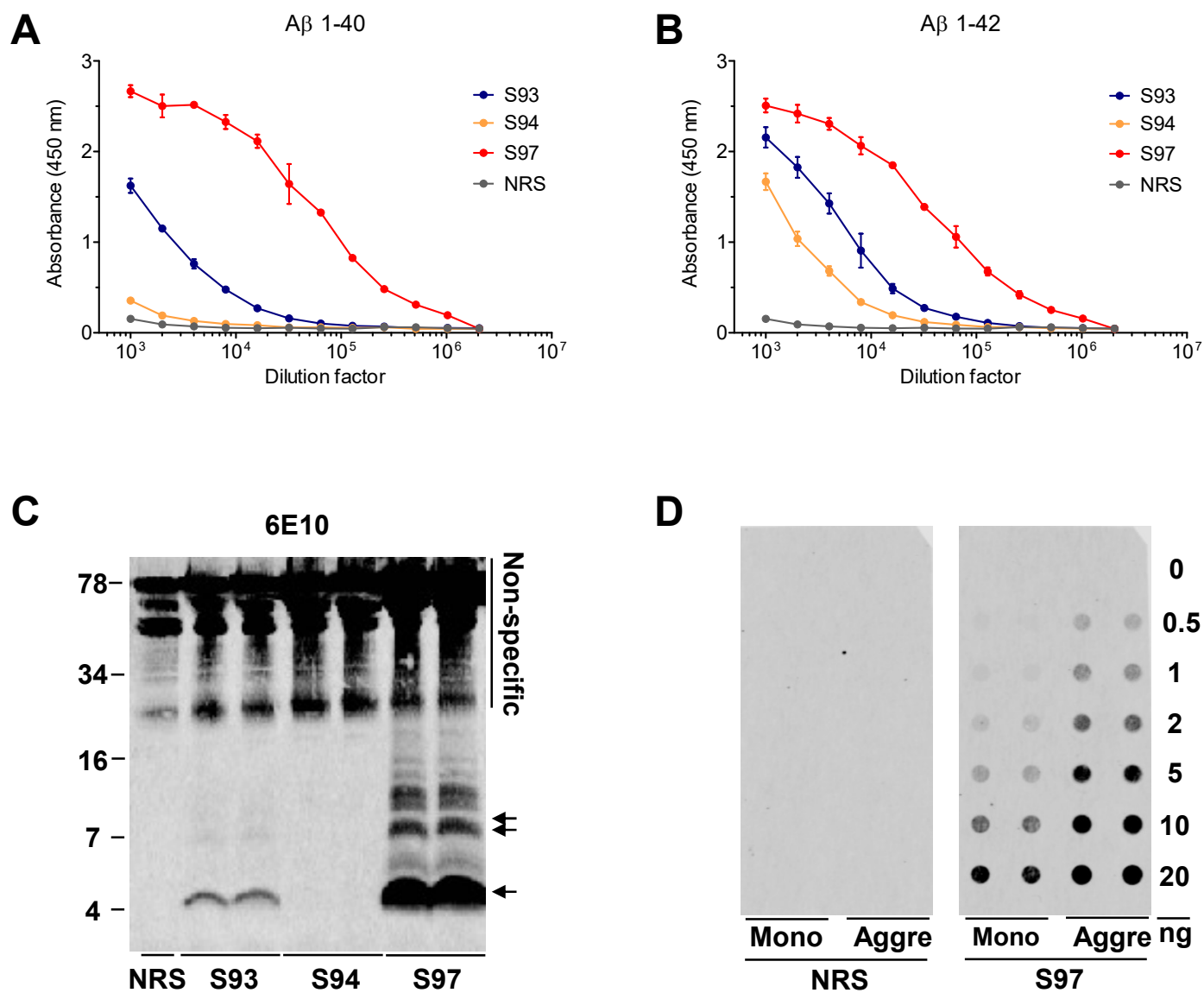

Supplement: Supplementary file 1 — Additional file 1: Fig. S1. Characterization of the novel anti-Aβ antiserum S97.Three rabbits (S93, S94 and S97) were repetitively immunized with aggregated synthetic Aβ1-42 and blood collected 1 week after each immunization. After the 4th immunization, all 3 rabbits produced detectable levels of anti-Aβ antibodies. Data shown are for sera collected after 7th immunization. (A) Aβ1-40 or (B) Aβ1-42 were immobilized on ELISA plates at 30 ng/well and serial dilutions of each antiserum or normal rabbit serum (NRS) added to the plate and detected with horseradish peroxidase (HRP)-conjugated secondary antibody. At dilutions in excess of 1 in a 100,000, only S97 still detected synthetic Aβ. (C) Using our well-established IP/WB protocol, the same 3 antisera were tested for their ability to immunoprecipitate Aβ species from the conditioned medium of 7PA2 CHO cells [53]. Samples were immunoprecipitated using S93, S94, S97 and NRS and Western blotted with 6E10. Immunoreactive Aβ-specific bands migrating at ~4 and ~8-10 kDa are indicated with arrows. Non-specific bands detected in samples exposed to NRS are indicated. S97 readily immunoprecipitated a range of Aβ species from 7PA2 conditioned media consistent with the pattern seen with other high affinity anti-Aβ polyclonal antibodies [43]. (D) SEC-isolated synthetic Aβ1-40 monomer and aggregated synthetic Aβ1-42 were dotted onto 0.2 μm nitrocellulose at the concentrations shown and detected with either S97 or NRS. S97 readily detected both monomeric and aggregated Aβ. [file 40478_2023_1511_MOESM1_ESM.pdf]

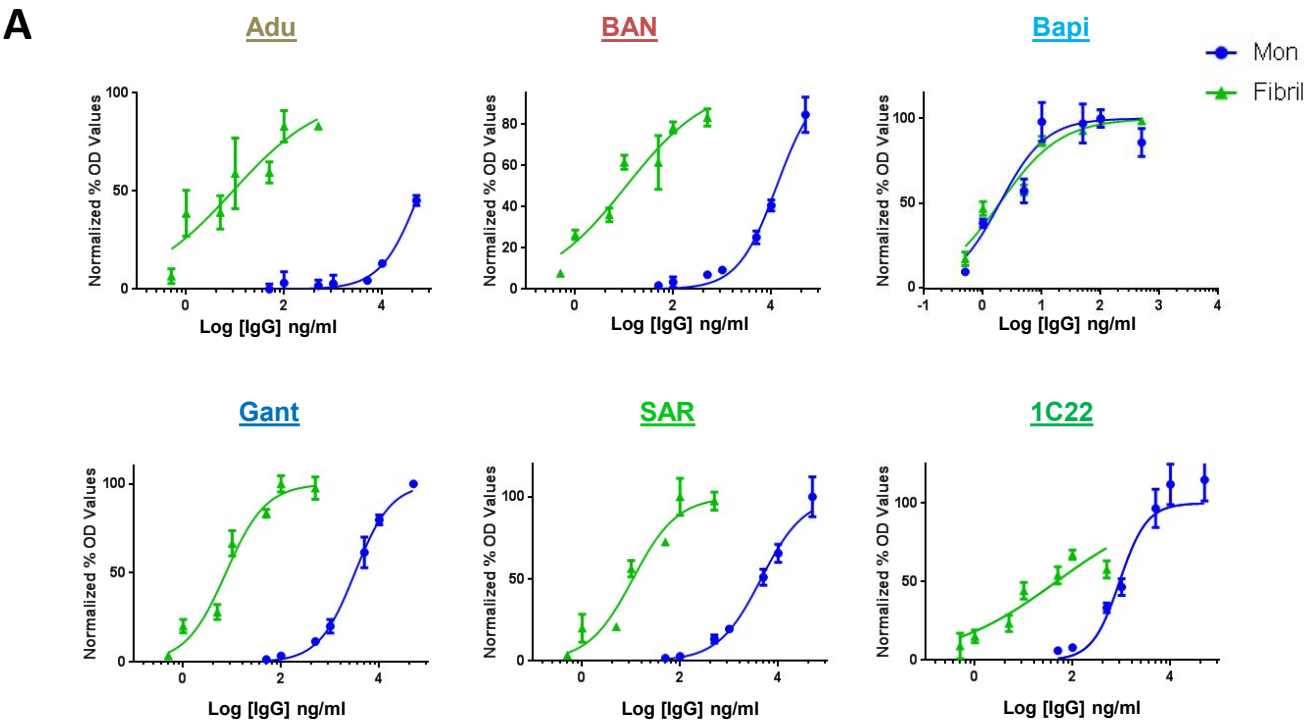

**B**

| Antibody                         | Adu  | BAN  | Bapi | Gant | SAR  | 1C22 |
|----------------------------------|------|------|------|------|------|------|
| Log EC <sub>50</sub> for Monomer | 5.36 | 4.25 | 0.48 | 3.3  | 4.41 | 3.43 |
| Log EC <sub>50</sub> for Fibril  | 2.48 | 0.58 | 0.39 | 1.25 | 1.9  | 1.79 |

Supplement: Supplementary file 2 — Additional file 2: Fig. S2. Most anti-Aβ antibodies preferentially recognize Aβ fibrils vs. monomers. (A) Plates were coated with 2.5 µg/mL of anti-Aβ antibody 4G8, Aβ samples applied, and test mAbs serially diluted across plates. Aducanumab (Adu, yellow), BAN2401 (BAN, red), bapineuzumab (Bapi, light blue), gantenerumab (Gant, dark blue), SAR228810 (SAR, light green) and 1C22 (dark green). OD values are normalized relative to Bapi, which was included in each plate. (B) Antibody binding EC50 values were calculated using a four-parameter, non-linear regression analysis of log concentration versus normalized OD. [file 40478_2023_1511_MOESM2_ESM.pdf]

SFigure 4

A

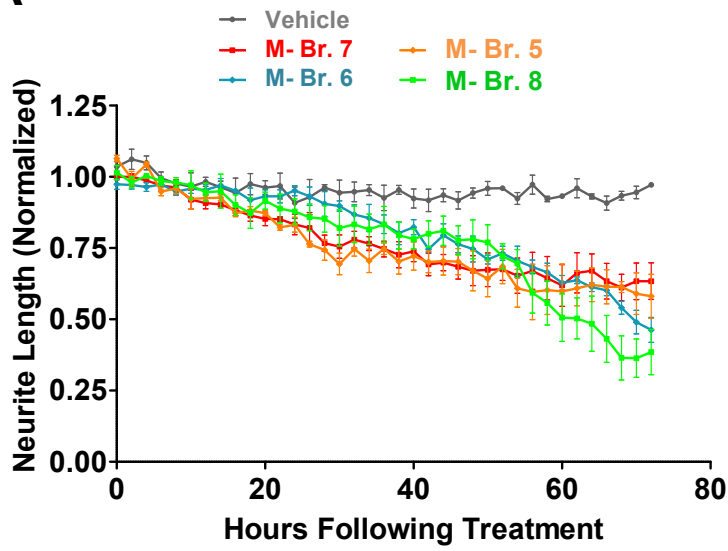

B

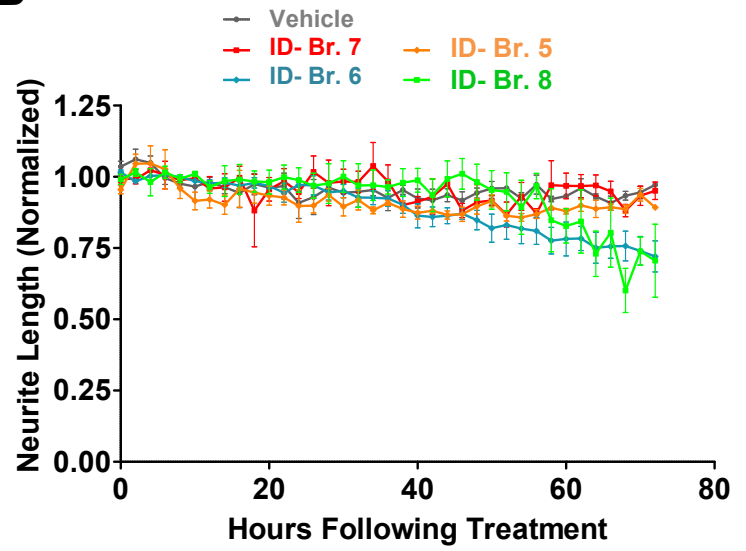

Supplement: Supplementary file 4 — Additional file 4: Fig. S4. Aqueous extracts of most AD brains are neuritotoxic. This Figure is an extension of Figure 1B and shows results for brain extracts Br.7, Br.6, Br.5 and Br.8. (A) iNs were treated with medium, mock-immunodepleted (Mock ID) AD brain extracts, or (B) extracts immunodepleted of Aβ with S97 (ID, right panel). Each well of iNs was imaged for 6 hours prior to addition of sample and NeuroTrack-identified neurite length calculated. Mock-ID and ID were tested at 1:4 dilution and cells treated with medium alone were used to monitor the integrity of untreated cells. Values are the average of triplicate wells ± SEM. [file 40478_2023_1511_MOESM4_ESM.pdf]

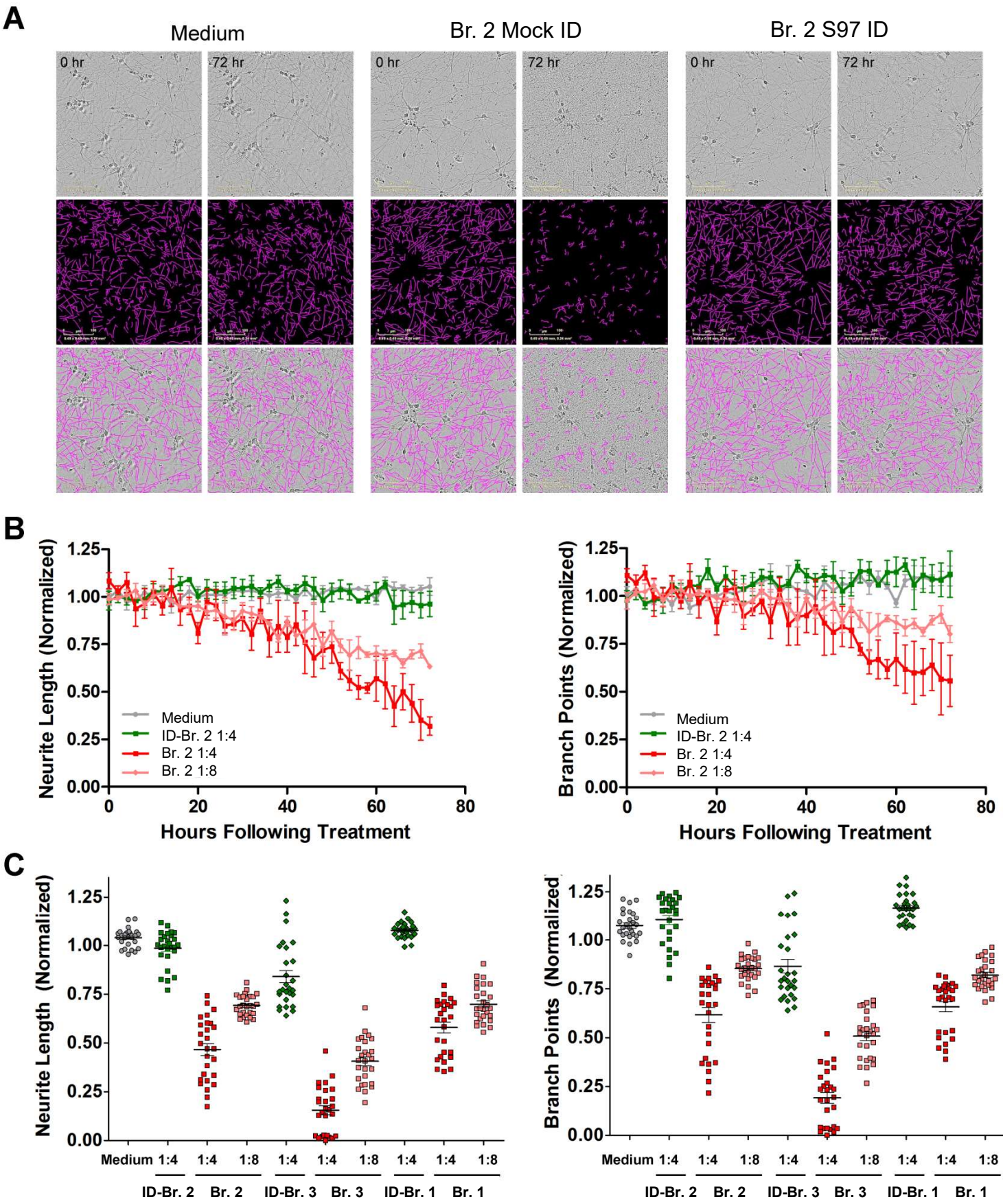

Supplement: Supplementary file 5 — Additional file 5: Fig. S5. Bioactive AD brain extracts dose-dependently induce neuritotoxicity. Live-cell imaging was used to monitor the effect of an Aβ-containing AD brain extracts on iNs. (A) iN day 21 cultures were treated with medium, or mock-immunodepleted (Mock ID) BR.2 or BR.2 extract immunodepleted of Aβ with the pan anti-Aβ antiserum S97 (S97 ID) and cells were imaged for 72 hours. Phase contrast images (top panels) at 0, and 72 hours were analyzed using the IncuCutye NeuroTrack algorithm to identify neurites (middle panels), and the NeuroTrack-identified neurites (pink) are shown superimposed on the phase contrast image (bottom panels). Scale bars are 100 μm. (B) Time-course plots of neurite length (left panel) and branch points (right panel) of treatments as in A. Each well of iNs was imaged for 6 hours prior to addition of sample and NeuroTrack-identified neurite length and branch points determined and used to calculate normalized neurite length and branch points measured at each interval. Mock-ID extract BR.2 was tested at 2 dilutions, 1:4, and 1:8. ID-BR.2 was tested at 1:4 and cells treated with medium alone were used to monitor the integrity of untreated cells. Data points are the average of triplicate wells ± SEM. (C) Plots of normalized neurite length (left panel) and neurite branch points (right panel) for the last 9 time points are shown as mean values ± SEM; i.e., a total of 27 data points per treatment. Brain extracts Br.2, Br.3 and Br.1 caused dose-dependent neuritotoxicity, whereas the same extracts immunodepleted of Aβ had no effect. For neurite length measurement, ID-Br.2 vs. medium, p=0.87, Br.2 1:4 vs. medium, p<0.0001, Br.2 1:8 vs. medium, p<0.0001; ID-Br.3 vs. medium, p=1, Br.3 1:4 vs. medium, p<0.0001, Br.3 1:8 vs. medium, p<0.0001; ID-Br.1 vs. medium, p=0.93, Br.1 1:4 vs. medium, p<0.0001, Br.1 1:8 vs. medium, p<0.0001 (Two-way ANOVA test). For branch points measurement, ID-Br.2 vs. medium, p = 1, Br.2 1:4 vs. medium, p<0.0001, Br.2 [file 40478_2023_1511_MOESM5_ESM.pdf]

SFigure 6

**A**

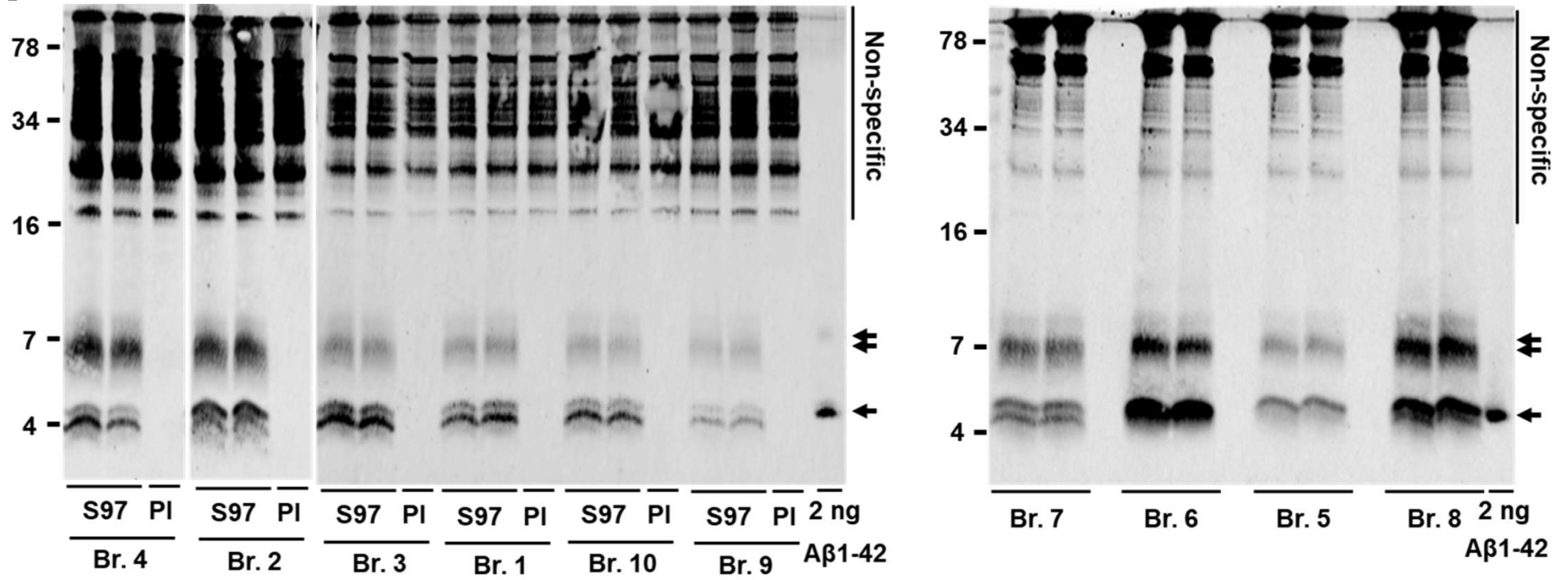

**B**

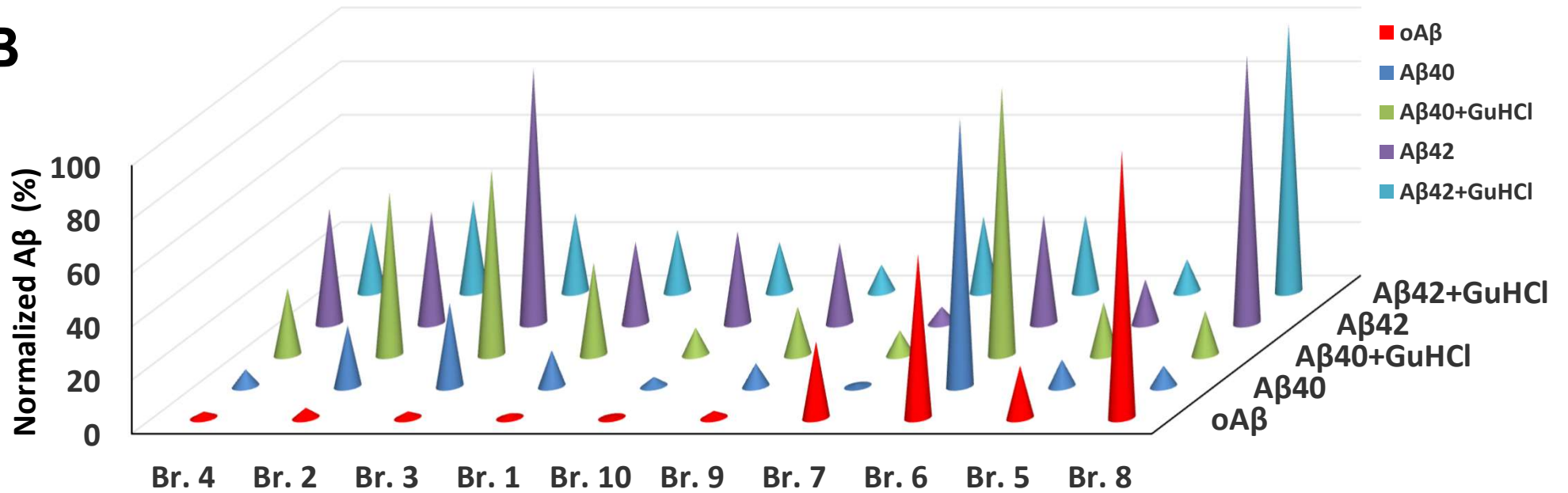

Supplement: Supplementary file 6 — Additional file 6: Fig. S6. The water-soluble extracts of AD brains contain a mixture of Aβ monomers and soluble aggregates. Brain extracts were analyzed using 6 distinct approaches. (A) Samples were immunoprecipitated using S97 or preimmune serum (PI) and western blotted with combination of 2G3 (to Aβ40) and 21F12 (to Aβ42). Immunoreactive Aβ-specific bands migrating at ~4 and ~7 kDa are indicated with arrows. Non-specific bands detected in samples treated with PI are indicated. (B) Aβ monomer levels were measured using MSD-immunoassays that recognize Aβ40 and Aβ42. An assay that preferentially detects soluble aggregates (oAβ) was used to measure soluble aggregates in their native state. In an orthogonal approach, samples were pre-treated with 5 M GuHCl to disassemble soluble aggregates and the resulting Aβ40 and Aβ42 monomers were detected using the MSD Aβ40 and Aβ42 immunoassays. For IP/WB, samples were analyzed in duplicate, whereas samples were analyzed in triplicate for MSD-immunoassays. Values for MSD immunoassays are normalized to the brain extract which contained the highest amount of Aβ for a given analyte. The results shown are representative of 3 independent experiments. The raw data used to generate the graph in B are provided in Fig. 3. [file 40478_2023_1511_MOESM6_ESM.pdf]

A

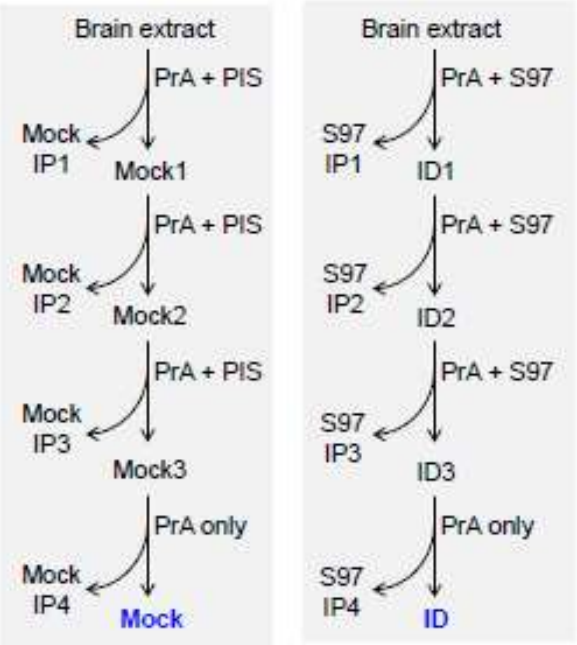

B

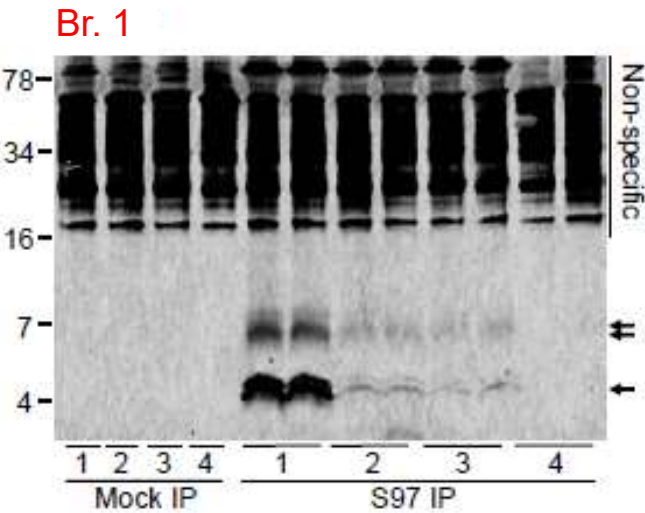

C

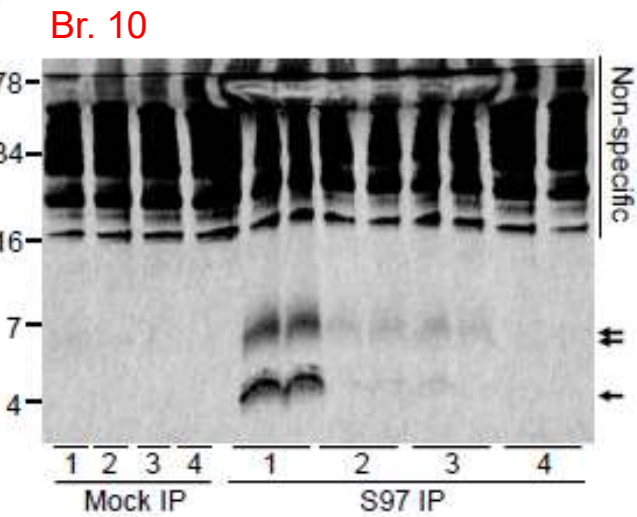

D

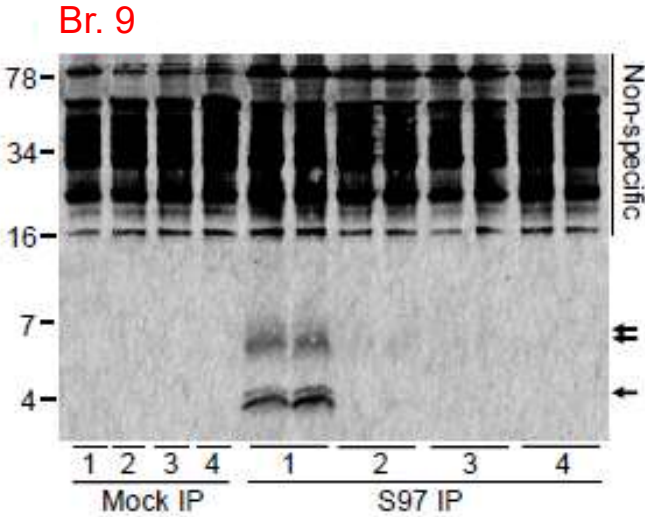

Supplement: Supplementary file 7 — Additional file 7: Fig. S7. The S97 pan anti-Aβ polyclonal antibody effectively depletes Aβ from AD aqueous extracts. Samples were immunoprecipitated using S97 or preimmune serum (PI) and protein A sepharose (PrA) for three rounds and an additional mop up step with PrA only. (A) Workflow of S97 immunodepetion (right panel) and mock immunodepletion (left panel). (B-D) Representative blots of IP’d materials from AD brain Br.1, Br.10 and Br.9. Aβ-specific bands were visualized with a combination of 2G3 (to Aβ40) and 12F12 (to Aβ42). Aβ monomers and dimers are indicated with single and double arrows. Non-specific bands detected in samples are indicated on the right. [file 40478_2023_1511_MOESM7_ESM.pdf]

A

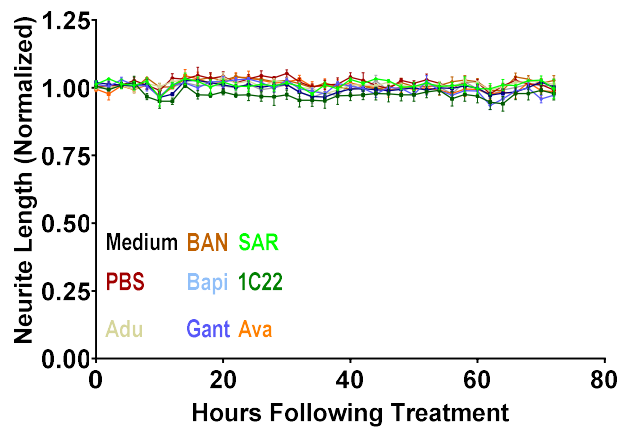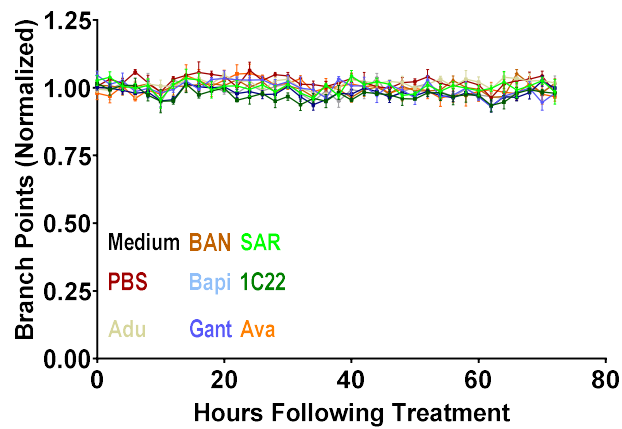

B

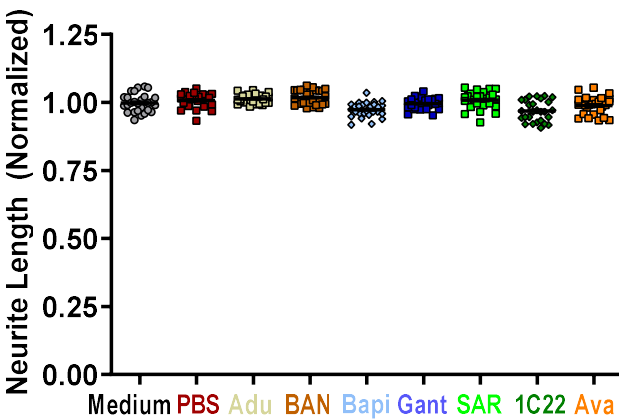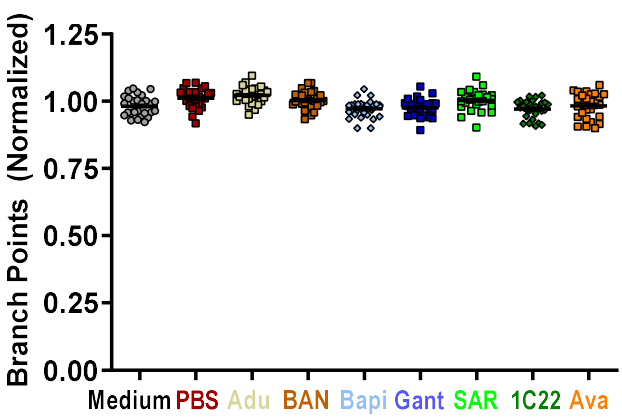

Supplement: Supplementary file 8 — Additional file 8: Fig. S8. Anti-Aβ mAbs alone do not alter neurite integrity. (A) iN day 21 cultures were treated with medium, or PBS (1:100 dilution) or antibody (1:100 dilution in PBS, 3 μg/mL) and cells were imaged for 72 hours. Each well of iNs was imaged for 6 hours prior to addition of sample and NeuroTrack-identified neurite length and branch points determined and used to normalize neurite length (left panel) and branch points (right panel) at each interval. The values shown in graphs are the average of triplicate wells for each treatment ± SEM. (B) Plots of normalized neurite length (left panel) and neurite branch points (right panel) are derived from 3 wells over the last 9 time points and are presented as mean values ± SEM. [file 40478_2023_1511_MOESM8_ESM.pdf]

SFigure 9

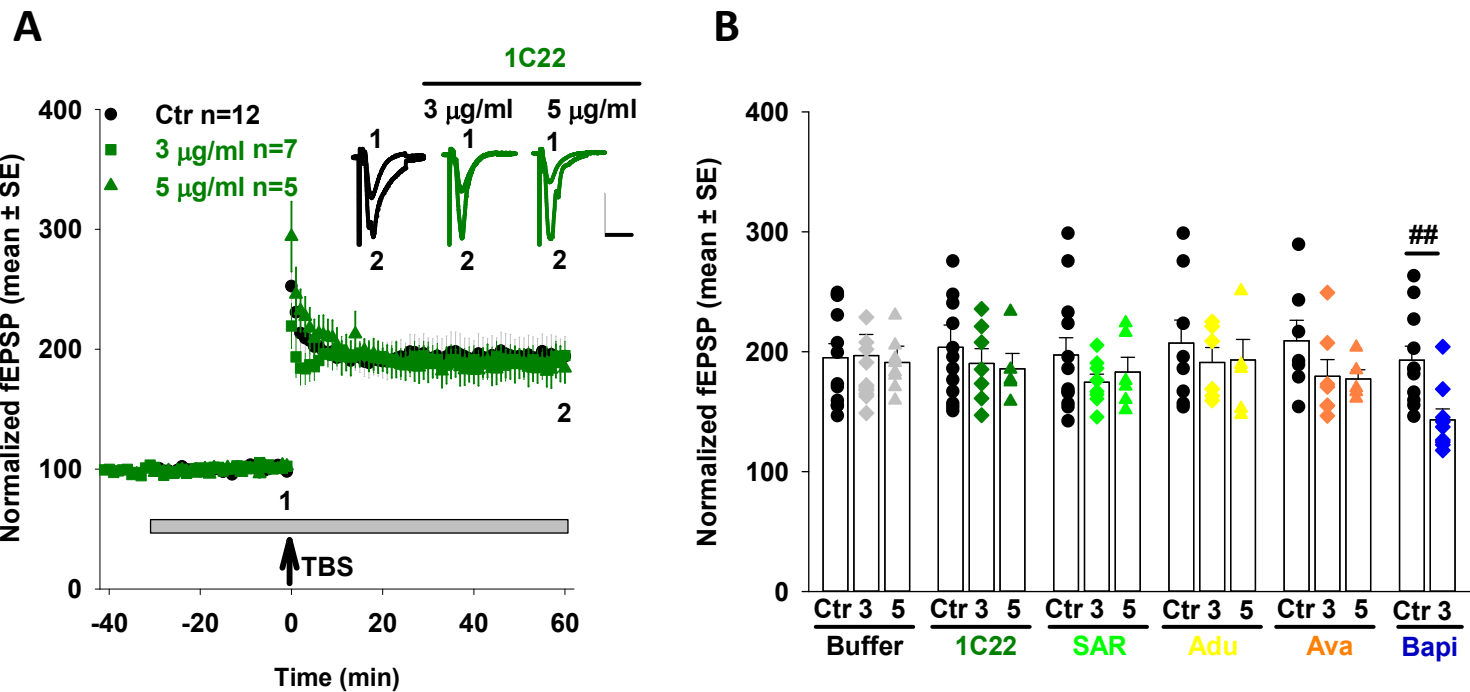

Supplement: Supplementary file 9 — Additional file 9: Fig. S9. Bapineuzumab but not other anti-Aβ mAbs affect LTP. (A) An example time course plots show that 1C22 alone had no effects on hippocampal basal neuronal transmission and LTP. aCSF control is shown using black circles; 3 µg/mL 1C22 treatments are shown using dark green squares; and 5 µg/mL 1C22 treatments are shown as dark green upward pointing triangles. The gray horizontal bar indicates the time period when sample was present in the bath. 1, 2, indicate example traces from time points just prior to the theta burst stimulation (↑ TBS) (1) and 60 minutes after TBS (2), respectively. Each slice used for each treatment was from a different animal. Scale bar 0.4 mV, 10 ms. (B) Histogram plots of the average potentiation for the last 10 minutes of LTP recording treated with 5 different mAbs and vehicle buffer (10 mM Histidine in 8% sucrose, pH 6.0) at 3 and 5 µg/mL. Note in order to maintain blinding of samples, 3 separate controls were tested, 2 of which are designated 3 and 5, respectively. Compared to aCSF control, vehicle buffer, 3 comparative mAbs (IC22, SAR and Adu) and control antibody (Ava) had no effects on LTP (Ctr n=15 vs. 3 µg/mL buffer n=12, F=4.3，p=0.93; Ctr n=15 vs. 5 µg/mL buffer n=8, F=4.41，p=0.72; Ctr n=12 vs. 3 µg/mL 1C22 n=7, F=4.45，p=0.67; Ctr n=15 vs. 5 µg/mL 1C22 n=5, F=4.54，p=0.55; Ctr n=12 vs. 3 µg/mL SAR n=8, F=4.41，p=0.24; Ctr n=12 vs. 5 µg/mL SAR n=6, F=4.49, p=0.53; Ctr n=8 vs. 3 µg/mL Adu n=6, F=4.75，p=0.53; Ctr n=8 vs. 5 µg/mL Adu n=6, F=4.75，p=0.61; Ctr n=7 vs. 3 µg/mL Ava n=7, F=4.75，p=0.2; Ctr n=7 vs. 5 µg/mL Ava n=5, F=4.96，p=0.17; One Way ANOVA test), whereas Bapi produced significant depression of LTP at 3 µg/mL (Bapi n=9 vs. Ctr n=11, F=4.41, p=0.005, One Way ANOVA test). aCSF control is in black circles; 3 µg/mL treatments are shown using squares and 5 µg/ml treatments with upward pointing triangles. ##p<0.01. [file 40478_2023_1511_MOESM9_ESM.pdf]

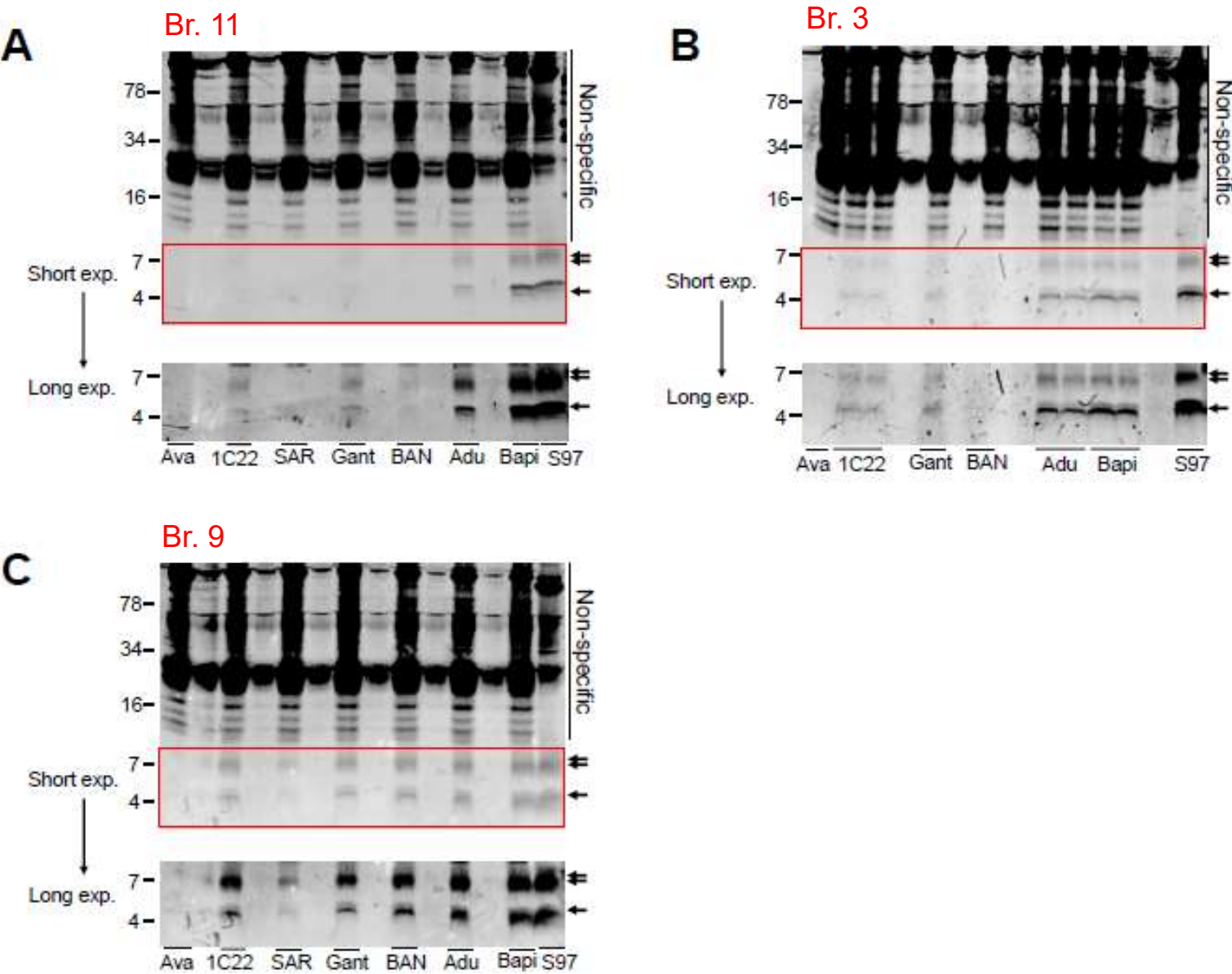

Supplement: Supplementary file 10 — Additional file 10: Fig. S10. Anti-Aβ mAbs immunoprecipitate Aβ from AD aqueous extracts. Half milliliter aliquots of Br. 11 (A), Br.3 (B) and Br.9 (C) brain extracts were immunoprecipitated using the indicated anti-Aβ antibodies along with 10 μL of 1:1 protein A Sepharose beads:protein G agrose beads. Ava and S97 were used as negative and positive control, respectively. Aβ-specific bands were visualized using a combination of 2G3 (to Aβ40) and 12F12 (to Aβ42). The insert below each full-length blot is an image of cropped portion of the same blot, but at longer exposure. Aβ monomers and dimers are indicated with single and double arrows. Non-specific bands detected in samples are indicated on the right. [file 40478_2023_1511_MOESM10_ESM.pdf]
