# Supplementary material for: Learnings about Aβ from human brain recommend the use of a live-neuron bioassay for the discovery of next generation Alzheimer’s disease immunotherapeutics
Source: Acta Neuropathol Commun. 2023 Mar 10;11:39. doi: 10.1186/s40478-023-01511-2 (PMC10007750; doi:10.1186/s40478-023-01511-2)
Supplement: Supplementary file 3 — Additional file 3: Fig. S3. Surface plasmon resonance reveals that all anti-Aβ antibodies preferentially recognize soluble aggregates over monomers. (A) Anti-Aβ antibodies were captured onto Protein A sensor chips and Aβ protofibrils or monomer flowed over the chip and binding assessed. (B) Kinetics data for anti-Aβ antibodies binding to protofibrils (PFs) and Aβ1-42 monomer (Mon). ND indicates that reliable estimates could not be determined. [file 40478_2023_1511_MOESM3_ESM.pdf]

SFigure 3

A

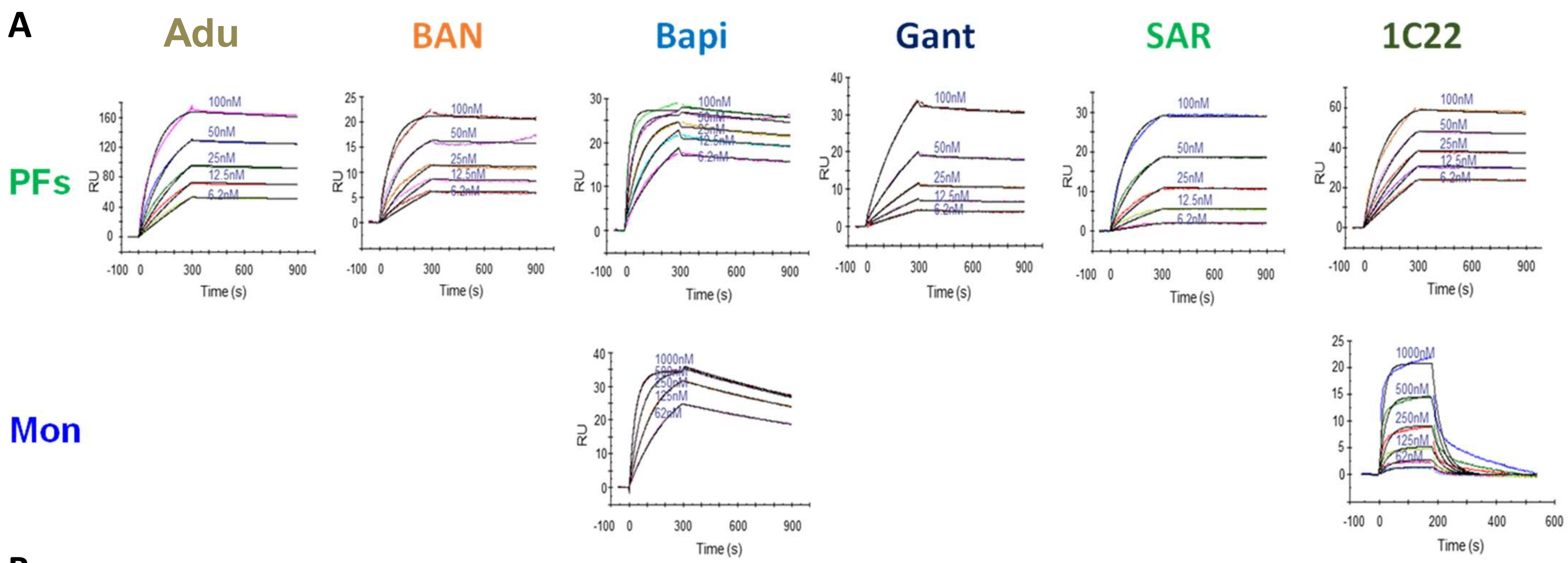

B

Antibody	Adu	BAN	Bapi	Gant	SAR	1C22
Profibrils binding mean K_D(nm)	0.12±0.04	<0.1±0.00	<0.1±0.00	0.81±0.35	0.43±0.57	<0.1±0.00
Monomer binding mean K_D(nm)	ND	ND	17±17	ND	ND	2120±2030
